# Current View of Nanoindentation: Recent Developments and Application in Material Characterization

**DOI:** 10.1021/acsami.5c05434

**Published:** 2025-06-11

**Authors:** Oleksandr Pshyk, Olesya Nakonechna, Emerson Coy

**Affiliations:** † 28501 Empa, Swiss Federal Laboratories for Materials Science and Technology, Ueberlandstrasse 129, 8600 Dübendorf, Switzerland; ‡ 231834 Institute of Magnetism of the NAS of Ukraine and MES of Ukraine, 36-b Vernadsky Blvd., 03142 Kyiv, Ukraine; § NanoBioMedical Centre, 529746 Adam Mickiewicz University, Wszechnicy Piastowskiej 3, 61-614 Poznan, Poland

**Keywords:** functional nanostructures, thin-film coatings, mechanical properties, electrochemical interfaces, combinatorial materials science

## Abstract

The first demonstration of qualitative nanoindentation several decades ago sparked enormous research on different types of materials, revealing unprecedented mechanical phenomena and properties. Since mechanical properties are directly linked to many materials’ structural and compositional features, nanoindentation has been successfully utilized to explore the coupling between mechanical behavior and functional properties, like electrical conductivity, reaction kinetics in electrodes, and hydrogen charging. Nanoindentation measurements have evolved beyond probing simple bulk metals, extending to complex alloys, nanomaterials, and nanocomposites. This review summarizes recent advancements in the nanoindentation technique, which now expands beyond traditional hardness and Young’s modulus measurement. Beginning with the fundamental mechanics and principles of indentation, we explore recent methodological developments. We discuss the interpretation and significance of key mechanical characteristics derived from nanoindentation including elastic strain to failure, resistance to plastic deformation, and elastic recovery. Furthermore, we highlight advanced approaches such as topographic reconstruction of thin films, combinatorial nanoindentation, and *in situ* and *operando* nanoindentation.

## Graphical abstract

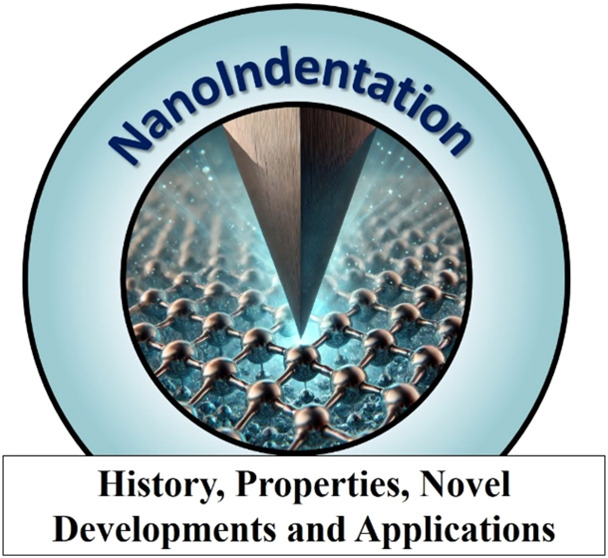

## 1 Introduction

With the great interest that both the academy and the industry have developed toward nanotechnology in the last 20 years, many characterization techniques have been forced toward new applications and research fields. Year by year, new materials, structures, and architectures are spotlighted in renowned journals due to their novel and surprising applications. Indentation, a well-known mechanical and functional characterization technique for materials, is not the exception among those techniques. Nanoindentation, as currently known, is, to a certain extent, the latest member of the available mechanical testing techniques. This is, of course, related to the bloom mentioned above of nanotechnology but is also a clear indication of its maturity. Despite common misconceptions, the mechanical properties of materials can and will determine their ultimate applicability and success in a given field. Many highly promising architectures have yet to see commercial exploitation, in significant part due to their poor mechanical resilience among other critical issues. The demand for more robust materials and devices is an ongoing cooperative endeavor between researchers and industrialists, aiming to characterize and understand the many fascinating possibilities that nanotechnology has provided. Therefore, this general review aims to introduce the reader, newcomers, and casual users to the general view of the nanomechanical testing of materials by nanoindentation and its applications in several fields. Several questions, such as What is it, What does it measure, and How does it work?, will be answered in detail and in a relatable manner. Finally, a few of the most representative applications of nanomaterials to date will be presented and some perspectives will be discussed.

The interest in the mechanical properties of materials could be as old as that of humanity itself. The old saying—the right tool for the right job—might as well include many properties of the “right tool”, and among them, definitively, its mechanical properties. Etymologically speaking, the word Indentation comes from the combination of two Latin words, *in* and *dents*, which could be roughly translated as “to mark with teeth”. This is of particular interest since it allows us to understand the origin and primitive attempts to qualitatively assess mechanical properties by using a relatively standard tool, our own teeth. When explaining the basics of nanoindentation and mechanical testing, many researchers and educators ask students, or the reader, in this case, a simple question. How hard is the wooden table in front of them? (Assuming there is one). This simple question has never failed to engage the person to reach out with their fingers to interact with furniture[Fn fn1]. Despite the time allowed for this experiment, the person typically remains clueless as to “how” hard the surface is. It is only when a simple rubber and a piece of solid metal are placed on the table that the person gets the idea that a comparative scale has been introduced. Then, the answers turn into a comparative sentence, in which the wooden table will be between the rubber and the metal piece if everything is according to nature.

In general, indentation techniques compare the mechanical strength of a given material and that of the model one, typically the hardest available. Usually, the resistance to deformation of a given set of materials can be evaluated by systematically pressing them against a harder one. The depth of the residual imprint on the tested materials could be used to sort them from soft to harder qualitatively. However, one can clearly see the many possible sources of error or dispute in this frugal introduction to Indentation. To mention the most critical and perhaps evident ones: (i) Similar to the issues between the imperial and metric systems, comparing teeth, nails, and stone imprints will be catastrophic, confusing and pointless. (ii) The depth of the imprints will strongly depend on the probing material’s shape, a peak that will do a great job, and the animosity of the lab technician (force). (iii) The visibility and clarity of the imprints vary depending on the surface of the material. A smooth surface is ideal. Finally, the one that could be the most critical of them all, (iv) is the hardness of the reference material. It should not encounter a harder one.

Despite the multiple technical aspects and standardization measures to be addressed in a nanoindentation experiment, the reader can be sure of a few things to this point. In order to evaluate the mechanical response of a sample, a known force has to be applied to the material using a known harder material, and the resulting deformation should be recorded, examined, and compared. This line of thought, grossly oversimplified, lies at the very core of the mechanical measurement field; broadly speaking, it describes what Thomas Young explored more than 200 years ago and, to this day, holds his name: The Young’s modulus (*E*).

### 1.1 What do We Measure? Short Historical Background

The Young’s modulus was investigated in 1807 by the versatile polymath Thomas Young, although it is known that other researchers, including the famous Euler, had fiddled with the topic much before. In essence, *E* appears as a normal consequence of Hooke’s law for springs. In simple terms, Hooke’s law (Figure 1a) describes that in a spring: the elongation (*x*) depends on the force applied to it (*F*) and its spring constant (*K*). Rewritten in mathematical terms and in a form that we can all relate to, it looks as follows: *F* = *Kx*. Notice that this equation is typically presented with a negative sign since it depicts hanged weights on a spring. The equation proposed by Hooke also shows one important thing: the force that spring will exert is proportional to its deformation (elongation), as many of us might have experienced during the breaking of a rubber band.

**Figure 1:**
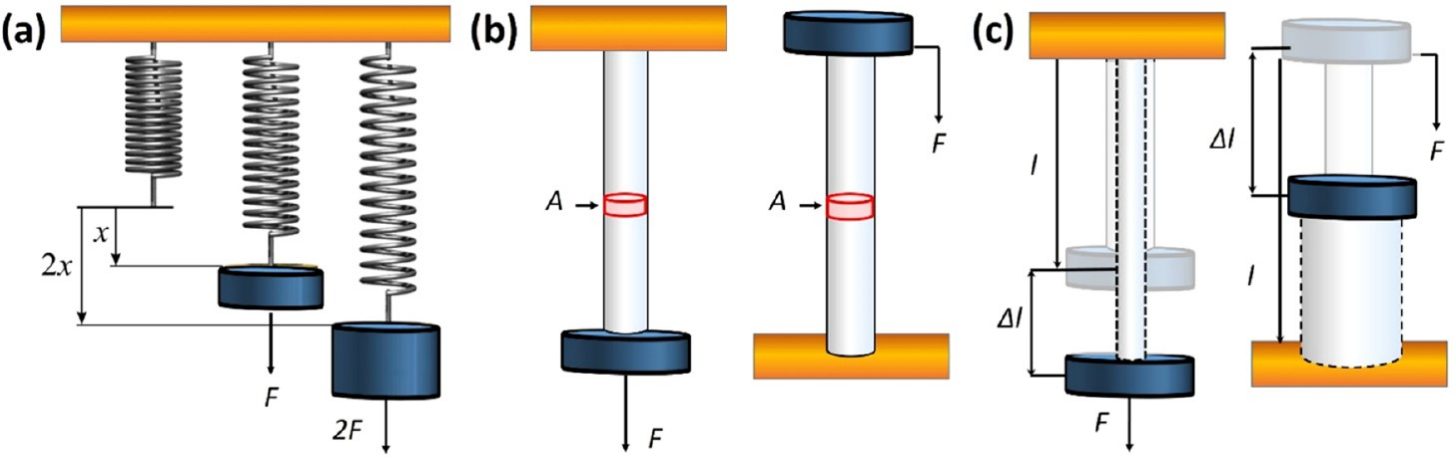
Schematic representation of (a) Hooke’s law. (b) Tensile and compressive stress (σ) and (c) tensile and compressive strain (ε).

It is important to remark that although the coiled spring has been around since the 15th century, and perhaps its behavior could be intuitively understood by many people, the inherent similarity between the spring and the mechanical response of materials was not. This is because, although the mathematical formulas are similar, they describe different phenomena. The general relationship between both the spring constant and Young’s modulus is that, in essence, both are determined by the applied pressure and deformation of the sample. However, while *K* refers to the properties of an object (also known as stiffness), *E* describes the properties of a given material. This difference can be easily understood by picturing two coiled springs made of the same material but with different roll diameters and wire thicknesses. While the materials used for manufacturing both springs will have the same *E* in both cases, their *K* values will be different.

In order to further understand Young’s modulus, two terms have to be introduced: Stress (σ) and Strain (ε). Although both terms are indiscriminately used or abused in much of the English-speaking world to refer to stressful and difficult situations, they are not the same. On the one hand, σ can be defined as the amount of force applied (*F*) on a material over a specific unit area (*A*), also known as cross-sectional area, as follows: = *F*/*A*. Therefore, σ has units of newton (*N*) per meter squared, shown as follows: *N·m* ^–2^. However, it is typically shown as an entirely new unit of measurement, known as Pascal (Pa), with a relationship of 1 Pa = 1 N·m^–2^. Also, notice that *F* is considered uniaxial, meaning it is applied perpendicularly to the material, without any additional components (Figure 1b). On the other hand, ε is the response of a given material to σ. As expected, the shape will change after an external force is applied to a material. Therefore, the ε is understood as the change in length (Δ*l*), elongation or compression of the material, with respect to its original dimension (*l*), as follows: ε = Δ*l*/*l*. It is important to remark that ε is a dimensionless parameter, meaning that it has no associated units (Figure 1c). Finally, notice that depending on the direction of the applied force, both σ and ε, can be labeled as tensile or compressive.

Having introduced σ and ε, their relationship with *E* can be easily presented and explained. The *E* is defined as the ratio between σ and ε, as follows *E* = σ/ε. Notice that since σ has units of Pascals (Pa) and ε is dimensionless, the *E* also has Pa units, typically kilo Pascal (kPa) for polymers and soft materials and giga Pascal (GPa) for ceramics and metals. A simple way to visually understand the importance of *E* in material sciences comes from plotting the σ vs ε and analyzing the shape and slope of the curve for a given material. Figure 2a shows several examples of the behavior of different materials during this test. One of the first observations that resulted from this plot is that commonly known “hard materials”, such as metals, will change their dimensions (ε) much less than relative “soft materials”, like rubbers-like when the same stress (σ) is applied. Apart from the clear different slopes (*E*) of the plots, several other characteristics come to sight when larger σ values are applied. Figure 2b, shows the example of a metal bar that is being stretched (tensile stress). The first region is what is called the linear section of curve (1), since the σ and ε clearly follow a geometrical relationship. A sudden deformation jump is observed after reaching the material’s maximum deformation (2). The material leaves the so-called “elastic regime” and enters the “plastic regime”. After this point, the maximum strength of the material (4) is found; finally, the material breaks following the short inflection in the response (5).

**Figure 2:**
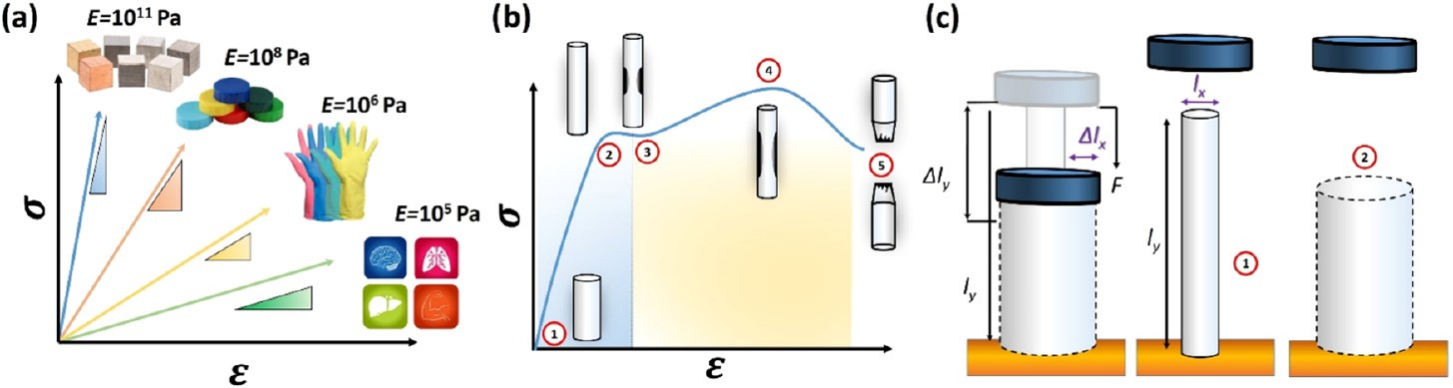
(a) Comparison between the visual behavior of the linear section of the *E* for different materials, from metals (10^11^ Pa), to plastics (10^8^ Pa), rubbers (10^6^ Pa), and biological tissues (10^5^ Pa). (b) General view of the full *E* plot showing the linear region (1) also known as the elastic regime. The yield point (2), the beginning of the plastic deformation on the material (3), the ultimate strength (4), and the fracture (5). (c) A comparison between elastic and plastic deformation; after removing the load (central image), the material returns to its original shape and dimensions (reversible deformation), and the sustained change of the material (irreversible deformation) after removing the load (right panel).

In order to understand the difference between the so-called plastic and elastic regimes, one can picture a similar example, in which a metal bar is subjected to uniaxial stress. Figure 2c, shows a similar case used before to understand σ and ε. After pressing the metal bar, this one reduces dimensionally, reaching a point of maximum deformation Δ*l*_*y*_. On the one hand, in a fully elastic material, or in the elastic regime of a given material, after removing the Load, the material will go back to its initial shape and dimension (1). Thus, this process is called reversible. On the other hand, if the material is already in the plastic regime, the new shape and dimensions will be retained after removing the Load (2); therefore, this process is irreversible. The attentive observer would have already noticed that in Figure 2c, a new distinction has been introduced; not only is the uniaxial strain shown, but also the one perpendicular to the direction to the applied force, labeled as *l*_*x*_ and Δ*l*_*x*_.

Here, it is essential to notice the importance of the discovery of rubber in the mechanical analysis of materials. Rubber, a substance endemic to the Americas and brought to Europe in the mid-18th century, might have influenced many changes in the general perception of materials. Before Young, most of the studies on the mechanical response of materials used their vibration, bending, or bouncing, to qualify such properties. Young had seen the importation and production of rubber and gums to Europe during his lifetime. Although they are widely confined to textiles, many scientists have experimented with bending properties and wave propagation. Thomas Young wrote in 1807, “We may easily observe that if we compress a piece of elastic gum in any direction, it extends itself in other directions; and if we extend it in length, its breadth and thickness are diminished”.^1^ Although this sentence was supposed to be illustrative, it awoke the curiosity of a French mathematician Siméon Denis Poisson, who later came up with yet another well-known ratio, the Poisson’s ratio (υ).^2^

The Poisson’s ratio describes an important aspect of materials under deformational or mechanical work.^3^ As Young had described, the changes in volume for an elastic gum are obvious but provide an important view on the constituents of the material. As presented in Figure 2c, when a material is compressed not only uniaxial ε_*y*_ is present in the sample, additionally a perpendicular ε_*x*_ appears as the material changes its shape. The general formulation for υ follows the following equation: υ = −ε_*y*_/ε_*x*_, the negative sign refers to the typical inverted response of the material, expanding in one direction and contracting in the other. Typical values for υ are between 0.1 and 0.5. The classic example of a material with almost zero value for υ is the wine cork. When a wine cork is pressed into the bottleneck, if its value is higher than, for example, the nominal for rubber υ = 0.5, the pressure applied on the cork makes it become considerably shorter and thicker, which will result in a rather catastrophic pressure to the glass bottle. Also, notice that once again, υ is dimensionless since it is the result of two dimensionless parameters, ε_*y*_ and ε_*x*_.

Finally, it is important to remember that a few other moduli exist, all resulting from Hooke’s law. Bulk modulus (*K*) represents a similar response as the one presented here for *E*; however, the applied force is uniformly distributed over the entire volume (i.e., hydrostatic pressure). The Shear Moludus (*G*, or sometimes *S*) refers to the deformation of a material when a side is subjected to a contrary force (i.e., friction).^4^ The latter is also important for the flow or transference of energy along materials (shear waves), making the *G* vital for acoustics, ultrasonic, and seismic studies.

### 1.2 How Do We Measure Nanoindentation?

Having introduced some of the history of the mechanical properties, of materials, there is the question of “*How to extract mechanical values from nanomaterials?*.” In fact, as mentioned in the introduction of this text, there are many issues for the accurate measurement of mechanical properties, especially when it comes to extrapolating contact methods from Hooke’s law. Among them, many are technological, i.e., the accurate application and recording of nanometric forces and displacements, but also methodological, i.e., the comparable and reproducible measurement of mechanical values from nanomaterial. Leaving aside the technological aspects, which will be presented in short later in this text, the main focus in this section will be the methodological and standardized aspects of the technique.

Nanoindentation relies on physical probing of a sample by a reference material. As early as 1859, scientists realized that by pressing a smooth material by another, a comparison of their mechanical response could be more accurately investigated.^5^ It is important to notice that before the aforementioned study materials were physically rubbed to each other to determine which one suffered damages or scratches and, thus, will be catalogued as the softer. In the first half of the 1900s, the method had become more sophisticated; a spherical ball was pressed with a known force on the surface of the sample and the imprint was visually evaluated. As expected, this method allowed to characterize the plastic regime and properties of many materials.^6^ However, several aspects are still unresolved. The most important of all was the uncertainty of the property being measured.

Material Hardness (*H*) is defined as an engineering property, much like stiffness. It does not directly respond to the material itself but to its internal configuration and arrangement (microstructure). For the main part of the 20th century, the differences between *H* and *E*, remained quite blurry, in many cases being used without care and interchangeably when referring to material properties. As in many developments, the industry had a special interest in investing and promoting many studies to standardize these evaluations. It was then when the Vicker’s indenter and the Brinell–Meyer’s[Fn fn2] indenter first appeared. In essence, both indentation methods used probing material. However, they differed in the shape of the indenter. The Vicker’s indenter was a 4-sided pyramidal tip, while Brinell–Meyers used a spherical probe. Most of the microstructural processes for hardening alloys or materials we know today were still in their infancy, let alone the experimental technique for evaluating these changes. Vicker’s and Brinell–Meyer’s Indentation gained rapid acceptance, especially in the industry. However, one main aspect was clear: values and measurements between the methods were often incompatible. The Brinell–Meyer’s hardness used the applied Load (*L*) divided by the spherical imprint on the material (*H*_B_ = Brinell’s) or the projected area (*H*_M_ = Meyer’s) as follows

1 $$H_{B}=\frac{2L}{\pi D^{2}\left[1-\sqrt{1-{(d/D)}^{2}}\right]}$$

2 $$H_{M}=\frac{4L}{\pi d^{2}}$$

where *D* is the diameter of the indenter and *d* the diameter of the projected area, as shown in Figure 3a, notice that eq 1, is to be multiplied by 0.102 if for the force to be given in Newtons and for 9.81 to represent the result in MPa, while eq 2 is commonly already presented in MPa.^7^

**Figure 3:**
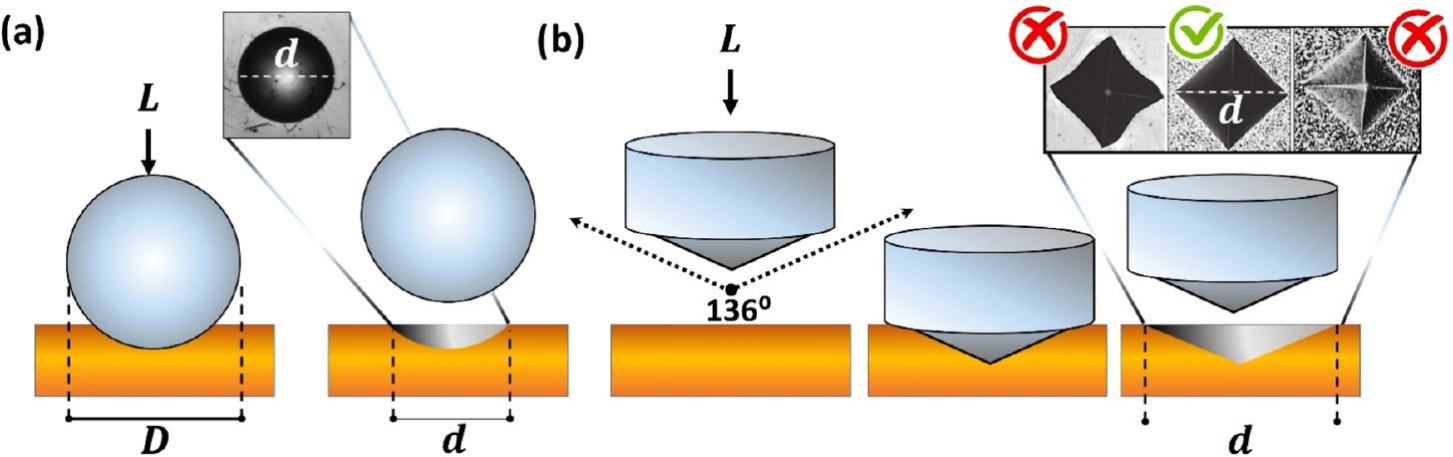
Schematic representation of (a) Brinell’s Indentation. The inset shows optical images of the residual imprint of a spherical indenter. *D* shows the total diameter, and *d* shows the reduced diameter of the imprint. (b) Vicker’s indenter from the angle of its indentation. The inset shows the residual imprint of the 4-sided pyramidal indenter, with 3 clear cases. Note that the imprint can easily identify artifacts on the measurement.

The approach proposed by Vicker’s was slightly different and appeared at a time when Brinell’s indenter was already quite known. By then, the Brinell’s test had been somehow standardized to leave imprinted marks between 0.2–0.5″, or 0.5–1.2 cm, in diameter. Thus, the average of those diameters was 0.375″ or 0.925 cm (also presented as 3/8″), which corresponds to an ascribed angle of 136°. Two aspects were addressed during this improvement. (i) a single indenter probe could be used for testing multiple materials and (ii) geometrical aberrations, such as nonperpendicular surfaces, can be easily identified as shown in Figure 3b. The general formula for Vickers’s hardness (*H*_V_) is presented in eq 3.

3 $$H_{V}=\frac{2L\sin(136^{{}^{\circ}}/2)}{d_{v}^{2}}=\frac{1.854L}{d_{v}^{2}}$$

where *L* is again presented in kg/force and needs to be multiplied by a similar factor as the Brinell’s hardness, 9.81, to be represented in MPa, and *d* represents the diagonal of the pyramid.^8^

Until now, the basics of Indentation used for most of the 20th century have been introduced. It is important to remark that Brinell’s and Vicker’s indenters rely on measuring mechanical imprints made on the samples. Therefore, the *H* values extracted from these methods are well in the plastic regime of the materials (Figure 2b), instead of in the linear section, where the *E* can be found. Despite this, implementing those standardized methods has allowed an unprecedented comparison and classification of mechanical values for different materials and applications. However, it became clear that in order to clearly evaluate the elastic regime of materials, not in tensile or compressive beam experiments but in real industrial and device conditions, a new theory or experiment should be explored.

In 1992, Warren C. Oliver and George M. Pharr published what would become one of the most iconic articles in materials sciences[Fn fn3]. Ian Sneddon’s studies strongly influenced the work in the late 1960s,^9^ who had been working on understanding the relationship between fully elastic interactions and the so-called contact depth (*h*_c_) and total displacement (*h*) problem. This problem appeared soon enough after the Vicker’s indenter gained popularity due to its improved shape and rather controllable indentation depth, allowing it to perform very small load tests. During the low dimensional test, most of the deformation induced in the samples was elastic, which meant that no plastic imprints were available for postoptical inspection. This particularity clearly pointed to the need to record the total displacement of the indenter in order to estimate its imprint magnitude. This is a pivotal moment in the history of indentation, since it led to the development of fully autonomous systems in which no human interaction or postoptimal measurements were needed.

In short, Sneddon investigated the fully elastic contact between two bodies and the implications for their contact depth. When a fully elastic contact is considered, the *h*_c_ and *h* are not the same because the curvature of the material’s surface reduces the contact between the bodies. It is important to remark that the study of this behavior gave birth to the field of Contact Mechanics and showed that when indents are performed in small dimensions, the elastic response of the indenter starts playing a more important role in the measurements.

When a spherical indenter is placed on an ideally flat surface, its curvature and diameter form what is called the initial separation distance *f*(*r*); this distance varies with the curvature of the indenter and can be predicted according to the diameter (*D*) of the indenter, as shown in Figure 4a. When a load *L* is applied to the system, the spherical indenter is pushed into the flat surface; because of the fully elastic interaction, the surface bends downward, and the contact with the sphere does not take place at its nominal thickness but at a reduced depth. Moreover, the general contact is dominated by the reduced diameter of the sphere, *d*, as shown in Figure 4b. The change in *f*(*r*) is therefore affected by the reduced diameter *d*, and the general separation by the curvature of the indenter can be defined as *f*(r*), which is the point when the reduced *d* and the contact with the spherical indenter are equal. Notice that this is a simplified form and that in the original Sneddon equation for spherical indenters, this is usually defined as *f*(*x*), where *x* = *r*/*a* and *a* stands for the contact diameter and *r* is the contact radius (*d*/2 in our case). For conical indenters Figure 4c, *f*(*x*) = (*a* cot­(Ψ))*x*, where Ψ stands for the angle of the indenter and *x* is a similar expression as for spherical indenter. Furthermore, when a conical indenter is considered, Figure 4c, the known geometry of the tip, along with the equations just described, allow for the determination of the *h*_c_ and *h* of a given indentation and, more importantly, lead to an accurate determination of the real contact area of an experiment.

**Figure 4:**
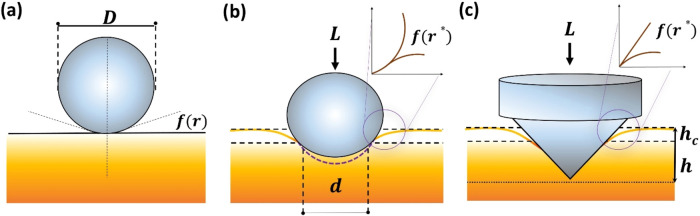
(a) Schematic representation of the interaction of a sphere with a flat surface. *f*(*r*) is the initial distance between the two bodies as a function of the sphere’s radius. (b) The contact when a sphere is pressed into the surface by load *L*. *d* represents the reduced diameter in contact with the surface, and *f*(r*) represents the change in the initial distance as the surface bends under deformation. Purple dashed lines show the ideal area the now elastically deformed sphere covers. (c) The case of a conical indenter pressed on a surface with contact depth (*h*_c_) and total displacement (*h*). *f*(r*) represents the distance as the surface bends under deformation.

Returning to the Oliver–Pharr method, let us know and discuss a common nanoindentation measurement. We know that a tip of known geometry is pressed against the surface of the sample. The tip is pressed into the material by applying incremental Load (*L*) while the tip’s displacement is recorded to determine the *h*_c_ and *h* the indent, Figure 5a. The typical experiment can be divided in three main stages (Figure 5b). First, the so-called loading phase (i) is defined as the pressing of the indenter on the material up to the maximum load (*P*_max_), and the second (ii) stage is the holding of the Load at the maximum penetration (*h*_max_) displacement. At this stage, the material being tested is allowed to allocate the applied stress. Finally, the unloading (iii) part of the experiment, records the “push” of the material toward the indenter, effectively the elastic response of the material. This last section is fitted by the modified Sneddon equation (eq 4), shown in Figure 5b.

4 $$S=\frac{dL}{dh}=\frac{2}{\sqrt{\pi }}E_{r}\sqrt{A}$$

where *S* is defined as the stiffness, *A* is the contact area between the indenter and the surface, and *E_r_* is the so-called reduced modulus. Notice that contact area *A* is intrinsically associated with both the indenter’s shape and the indentation test’s *h*_c_ and *h*.

**Figure 5:**
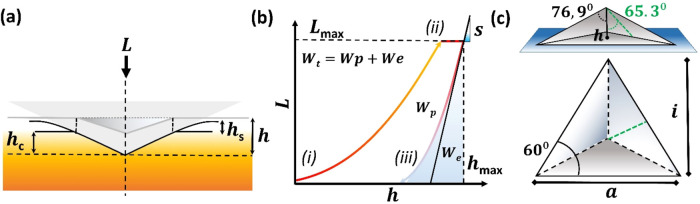
(a) Schematic representation of a nanoindentation experiment showing the *h*, *h*_c_, and the surface displacement at the contact perimeter (*h*_s_). (b) Three stages of Indentation, marked (i), (ii), and (iii), which are loading, holding, and unloading, respectively. The plot is presented in *L*(*h*), also known as load vs displacement. *L*_max_ stands for maximum load, *h*_max_ for maximum penetration, and *S* for the Sneddon equation. (c) Geometrical consideration of a Berkovich tip, view from the side (top panel) and from the top of the tip (bottom panel).

Up to this point, it must be clear that the contact area and shape of the indenter play crucial roles in the indentation experiment. So, it is important to introduce the most common shape found in nanoindenter instrumentation. One of the most used indenters today was first proposed in the early 1950s by Berkovich.^10^ This indenter tip quickly gained popularity as it brought several improvements to the field, among them, the simplicity of the tip, since it used a 3-sided pyramid instead of a 4-sided one like in the Vicker’s. This is important, mainly because, technologically speaking, it is much easier to build a sharp tip with three crossing planes than one with four planes. Additionally, due to the rather shallow shape of the pyramid and the clever geometrical arrangement of its shape, Figure 5c, the projected area (*A*_proj_) can be estimated only in terms of *h.* as shown in eq 5.

5 $$\begin{array}{l} A_{\mathrm{proj}}=\frac{a\cdot i}{2}=\frac{\sqrt{3}}{4}a^{2}\\ h=\frac{a}{2\sqrt{3\tan65.3^{{}^{\circ}}}}\\ A_{\mathrm{proj}}=3\sqrt{3h^{2}\tan^{2}}65.3^{{}^{\circ}}=24.45h^{2} \end{array}$$

where *a* refers to the length of the pyramid sides, *i* is the base to top height, and *h* the height of the pyramid, conveniently called in the same way as the total displacement of the indent. Therefore, the total indenter penetration can be expressed in terms of *h*, and by replacing eq 5 in eq 4, the only unknown term remains the reduced modulus (*E_r_*), which can be extracted from the experimental fitting. The projected contact area for Berkovich indenters is related purely to the indentation depth by a relatively simple power law assuming an ideal tip (no rounding or blunting), eq 5. However, for Vickers indenters, computation of the contact depth *h*_c_ via more complex correction is necessary for the determination of the contact area. This is achieved due to a self-similar, continuously sharp geometry of Berkovich indenters, in contrast to Vickers tips. Since Berkovich indenters remain self-similar and sharp at all indentation depths they maintain a predictable relationship between depth and contact area making them suitable even in elastic-dominated deformation. This is primarily because the sharp geometry of the tip ensures a localized stress field and because deformation is confined to a region that geometrically matches the tip shape. This along with a low tip imperfection impact at moderate depth makes Berkovich indenters better suited for materials with purely elastic response.

It is important to remark on the difference between *E* and *E_r_*. The reduced modulus comprehends the mutual elastic deformation of the indenter and the sample. As shown in Figure 4b, when small loads and penetration depths are used, the total deformation of both the indenter and the material becomes dominant. Despite this, the deformation is fully elastic at minimal loads and should be dominated by *E* of both the indenter’s tip and the sample. The general mathematical formulation of *E*_*r*_ is described as follows

6 $$\frac{1}{E_{r}}=\frac{\left(1-\upsilon \right)}{E}+\frac{\left(1-{\upsilon_{i}}^{2}\right)}{E_{i}}$$

Here, *E* and υ correspond to the Young modulus and the Poisson ratio of the sample, while *E*_*i*_ and υ_*i*_ represent the same values for the indenter’s tip. In this way, by knowing the υ for both the indenter and the sample and the *E* of the indenter, it is possible to extract the real *E* of the sample being examined. It is important to remark that this information has been previously discussed and outlined in several reviews and textbooks dealing with nanoindentation and mechanical aspects of materials, Table 1, and this information is compiled and presented in this review to help newcomers and the general public understand the section here contained, in a self-sustained manner. We did not intend this section to be an exhaustive review of the method but an approachable introduction to the topic; please refer to Table 1 for more detailed information.

**Table 1:** Other Literature Covering Important Aspects of Nanoindentation for the Reader’s Reference

title	refs
Instrumental Aspects	
accurate measurement of thin-film mechanical properties using nanoindentation	^11^
a review of nanoindentation continuous stiffness measurement technique and its applications	^12^
micro- and nanoindentation techniques for mechanical characterization of materials	^13^
Theory	
an improved technique for determining hardness and elastic modulus using load and displacement sensing indentation experiments	^14^
*In Operando* Nanoindentation	
*in situ* nanoindentation during electrochemical hydrogen charging: a comparison between front-side and a novel back-side charging approach	^15^
Combinatorial Characterization and Mapping	
nanomechanical property screening of combinatorial thin-film libraries by nanoindentation	^16^
high-throughput nanoindentation for statistical and spatial property determination	^17^
high-speed nanoindentation mapping: A review of recent advances and applications	^18^
*In Situ* XRD, TEM, SEM	
*In situ* characterization of stresses, deformation and fracture of thin films using transmission X-ray nanodiffraction microscopy	^19^
*In situ* nanomechanical testing in focused ion beam and scanning electron microscopes	^20^
*In situ* nanoindentation in the transmission electron microscope	^21^
Handbooks	
the IBIS Handbook of Nanoindentation	^22^
nanoindentation	^23^
Nanoindentation of Nanomaterials	
the mechanical properties of nanowires	^24^
*in situ* TEM nanoindentation of nanoparticles	^25^
determination of mechanical properties of carbon nanotubes and vertically aligned carbon nanotube forests using nanoindentation	^26^
AFM-based Nanoindentation	
some considerations in nanoindentation measurement and analysis by atomic force microscopy	^27^
nanotribology and Nanomechanics II	^28^

### 1.3 Nanoindentation-Derived Characteristics: *H*/*E*, *H* ^3^/*E* ^2^, *W*_e_

Over the years, hardness has been regarded as the prime mechanical property measured by nanoindentation used for mechanical properties characterization and an indicator of a material’s durability in a given technological application. It is well-known that many very hard materials, primarily ceramic thin films, are often very brittle, limiting their practical utilization. For practical engineering applications of such materials, their hardness should be accompanied by their toughness. It has been imperative for many years to achieve ultrahigh hardness of thin films. However, coating elasticity and toughness are equally important factors, particularly in abrasion, impact, and erosive wear. According to the definition, toughness is the ability of a material to absorb energy during deformation up to fracture, while fracture toughness is the ability of a material to resist the growth of a pre-existing crack. Therefore, toughness encompasses the energy required for the crack creation and further propagation until fracture, whereas fracture toughness accounts for the energy required to facilitate the crack propagation to fracture.^29^

Some theoretical and experimental studies^30,31^ suggest that the elastic strain to failure, which is related to the ratio of hardness and elastic modulus (*H*/*E*), can be a suitable parameter for the prediction of a material’s ability to resist mechanical degradation and failure. One reason why *H*/*E* was suggested as a proxy for toughness is that *H* is proportional to yield strength σ_*y*_ following the Tabor’s criterion *H* ≅ ασ_*y*_ ^32^ with 2.9 < α < 3.1 (α = 3 for metals). Therefore, *H*/*E* can be interpreted as the elastic strain limit and can be used as a measure of toughness. Moreover, as follows from the definition of the plasticity index given by Greenwood and Williamson^33^

7 $$\varphi =\frac{E}{H}{\left(\frac{\sigma }{\beta }\right)}^{1/2}$$

where σ is the surface roughness and β is the asperity radius, the mechanical contacts with small φ require large contact stresses to initiate a significant plastic deformation. This implies that a material with a large *H*/*E* is less likely to deform plastically at a given stress level, implying higher toughness when no plastic deformation exists. With the intention to reveal the role of elastic modulus in durability and wear resistance of hard protective coatings in hard disk applications, Tsui and co-workers^34^ introduced *H* ^3^/*E* ^2^ ratio, known as resistance to plastic deformation. It was done by adopting an analysis initially performed by Johnson^35^ for the estimation of the load needed to initiate plastic deformation, *P*_*y*_, when a rigid sphere of radius *r* is pressed into contact with an elastic/plastic half-space, also involving Tabor’s criterion

8 $$P_{y}=0.78r^{2}\frac{H^{3}}{E^{2}}$$

As follows from this equation, the contact loads required to initiate plasticity at a given test geometry are higher in materials with higher values of *H* ^3^/*E* ^2^, i.e., the likelihood of plastic deformation is reduced in materials with high hardness and low modulus. Although some pioneering works of Musil and collegues^36–39^ show a generally excellent correlation between *H*/*E* and *H* ^3^/*E* ^2^ ratios and the resistance to cracking during the bending of a wide range of nanocomposite thin films (Figure 6a), further utilization of these parameters to estimate the fracture toughness of materials in many publications appeared to be not appropriate.^40^ *H*/*E* and *H* ^3^/*E* ^2^ are attractive to use because *H* and *E* can be readily measured by nanoindentation, leading to many publications where both ratios are used to estimate fracture toughness. An accumulation of a large number of measurements of *H*/*E* and *H* ^3^/*E* ^2^ and fracture toughness for a diverse range of materials allowed to reveal that *H*/*E* and *H* ^3^/*E* ^2^are not generally applicable for the fracture toughness predictions and there is zero or inverse correlation with the fracture toughness is observed,^40^ Figure 6b. First, *H*/*E* and *H* ^3^/*E* ^2^ represent elastic strain prior to failure and resistance to plastic deformation and, thus, do not account for the contribution of plasticity to fracture toughness. Second, fracture toughness property does not depend on contact features. At the same time, *H* ^3^/*E* ^2^ is derived from surfaces in contact (eq 8). Before nanoindentation measurement, it is apriori not known if a material of interest contains any carriers of plasticity and can deform plastically. In contrast, most hard ceramics do not deform plastically at all. Therefore, *H*/*E* can be a good proxy for the fracture toughness of materials whose fracture toughness is dominated by elastic deformation only. It can also be used for the fracture toughness ranking of materials deforming beyond the yield point, while for materials deforming plastically without crack propagation, which does not lead to instant failure, the correlation does not hold. *H* ^3^/*E* ^2^ can be used as a measure of resistance to fracture initiation in brittle materials, which then can be taken as a measure of their toughness rather than fracture toughness. Although high *H*/*E* and *H* ^3^/*E* ^2^ ratios can be a measure of enhanced resistance to crack initiation, there is sufficient evidence confirming that they should not be considered as proxies for fracture toughness if material fracture involves plasticity. However, even when used, materials postdeformation and fracture events must be studied thoroughly for an accurate determination of whether plastic or elastic deformation dominates during nanoindentation.

**Figure 6:**
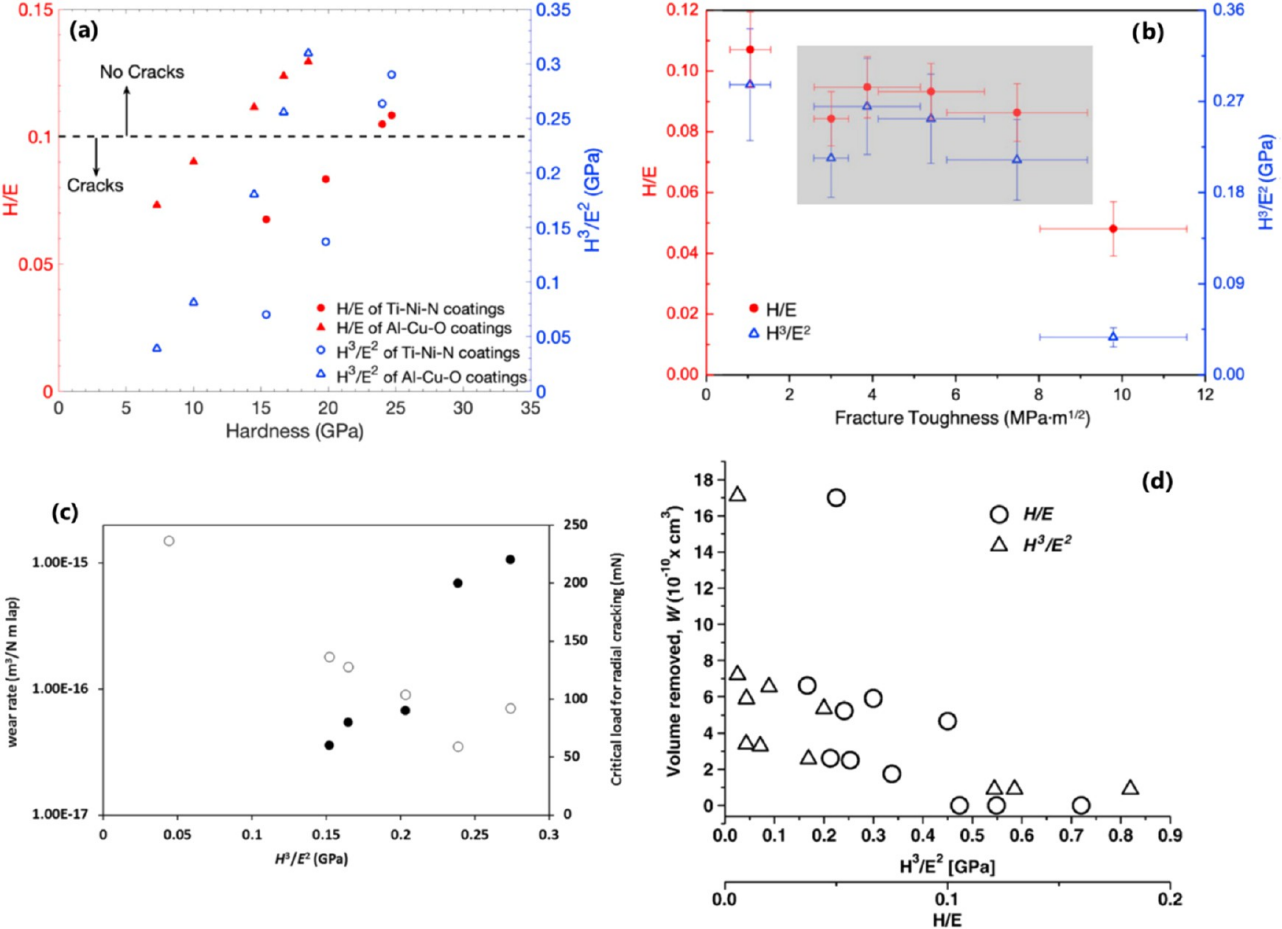
(a) Correlation between *H*/*E* and *H* ^*3*^/*E* ^2^ and the resistance to cracking during bending for Ti–Ni–N and Al–Cu–O thin film (data from refs 39 and 49 summarized by Chen et al.^40^) and (b) correlation between *H*/*E* and *H* ^*3*^/*E* ^2^ and fracture toughness (data from ref 50 summarized by Chen et al.^40^). (c) Correlation between *H* ^*3*^/*E* ^2^, wear rate (open circles), and critical load for radial cracking (closed circles) of *nc*-TiC/*a*-C:H nanocomposite coatings,^51^ taken from ref 42. (d) Relationship between the *H*/*E* and *H* ^*3*^/*E* ^2^ ratios and the volume removed from a coating under solid particle erosion.^45^ Taken with permission from refs 39,40,42,49–51 .

Since *H*/*E* and *H* ^*3*^/*E* ^2^ ratios correlate strongly with energy dissipation in mechanical contact, this has led to the exploitation of these ratios as proxies for wear resistance. Moreover, since many of the mechanisms of thin film failure during wear begin with or directly involve plastic deformation, it was proposed that thin films must be highly resistant to plastic deformation during contact events.^34^ Since the pioneering work of Tsai et al.^34^ and further developments in this field done by Leyland and Matthews,^30^ *H*/*E* and *H* ^*3*^/*E* ^2^ ratios of coating systems have been exploited over the last few decades for wear resistance estimation solely based on the determination of these two ratios.^41,42^ For instance, besides a strong correlation between the *H* ^*3*^/*E* ^2^ ratio and a critical load at which radial cracks formed when *nc*-TiC/*a*-C:H (*nc-* stands for nanocrystalline and *a-* stands for amorphous) nanocomposite coatings are indented with a Berkowich indenter, the coatings with higher *H* ^*3*^/*E* ^2^ ratio exhibit a much lower wear rate under dry sliding against hardened steel (Figure 6c). Wear rate is the amount of material removed from a surface due to mechanical action (volume loss per unit meter per unit load); in Figure 6c the wear rate is given m^3^/Nm. However, many other factors can impact the wear resistance of complex nanocomposite thin film and need to be taken into account prior to the utilization of *H*/*E* or *H* ^*3*^/*E* ^2^ ratios for any estimations of the damage tolerance under different tribological conditions for the design of efficient wear-resistant coatings.^42^ Therefore, these mechanical properties descriptors should be utilized with great attention when estimating wear resistance. It was recently revealed that *H*/*E* ratio markedly overestimates the wear resistances of strain-hardened metals.^43^ Furthermore, the interdependence of *H*/*E* and *H* ^*3*^/*E* ^2^ and the erosion rate of thin film materials has been demonstrated for many thin-film materials, which is especially relevant for hard protective thin films.^44^ It was revealed that the *H*/*E* and *H* ^*3*^/*E* ^2^ ratios should be maximized by reducing *E* while maintaining an optimal *H* to provide the best combination of plastic deformation resistance and brittle failure leading to low solid particle erosion (Figure 6c,d).^45^ More details about the limits of applicability of correlation between the *H*/*E* and *H* ^*3*^/*E* ^2^ ratios and coating wear resistance are given in the recent review papers with critical examination of experimental approaches/factors impacting an accurate determination of *H*/*E* and *H* ^*3*^/*E* ^2^ values by nanoindentation including best practice recommendations.^30,40,42^

Another parameter, easily extracted from load–displacement curves, is elastic recovery; *W*_e_ = *A*_1_/(*A*_1_ + *A*_2_), *A*_1_ and *A*_2_ are the elastic energy (the area under the unloading curve, Figure 5b) and the plastic (dissipated) energy, respectively, applied to the film during nanoindentation. *W*_e_ have been effectively used to characterize the elasticity of different nanocomposite thin films and their resistance to cracking and superelastic effects.^16^ Many nanocomposite thin films with very different *H* can exhibit the same elastic recovery *W*_e_ ^46^ that allows the design of superhard thin films demonstrating high elastic modulus up to the values of diamond, high hardness (>40 GPa), and high elastic recovery (*W*_e_ > 90%) combined with a high resistance against crack formation.^47,48^

## 2 Current Advancements in Applications

Finally, after this rather ambitious introduction and outline of the origins, initial challenges, and principal actors in the field of nanoindentation and mechanics, we have arrived at the point in which a fully automatic and reproducible method for mechanical analysis of nano/micro materials is available, and many companies based in these principles have produced highly advanced and competitive Nanoindenters and Tribometers.

### 2.1 Topographic Reconstruction of Thin Films and Mapping

Thin films are among the most common and versatile nanomaterials to date. They are used in many industrial applications, from electronic gates, contacts, hard drives, memories, and capacitors to protective coatings, functional bio surfaces, and even heat barriers. Thin films are made from a plethora of materials: ceramic, metallic, polymeric, or multielement composites (two-dimensional (2D) laminates or three-dimensional (3D) composites). The multiple architectures and combinations make the family of thin films a very rich and complex family to classify. However, all of these films share a classification characteristic. In order to be considered thin films, their thickness is typically ∼100 nm or less. The extremely low thickness poses an important challenge for the nanoindentation technique, in which many authors propose a minimum indentation depth equal to 10–20% of the total thickness of the film in order to provide reliable values. However, when very small thicknesses are evaluated, the substrate of the films brings a more dominant contribution than that of the film itself, making the evaluation of thin films a big challenge for nanoindentation.

One of the main problems in thin films is that the plastic deformation and stiffness response are coupled with those of the substrate up to a critical point; typically, these artifacts are not observed when *P*_max_ is below 15–20% of the total thickness of the film. However, different materials with high elasticity can prove to be quite difficult to evaluate since penetration can be too low, thus rendering it impossible to extract the true hardness of the film or too large, so the influence of the substrate becomes dominant.

A methodology for solving this issue is closely bound to the Oliver and Pharr method,^14^ and was initially described by Korsunksy et al.^52^ The model considers the total displacement of an indent on the film and the substrate with progressively higher indentation *L* values. In other words, the model considers the existence of a transition region between film and substrate, which can be predicted and extrapolated from experimental data. According to eq 7, gradually increasing the indentation loads can represent both the mechanical properties of the sample and the substrate (Figure 7a). The following equation represents this approach.

9 $$H_{c}=H_{s}+\frac{H_{f}-H_{s}}{1+{\mathrm{kh}}^{2}}$$

In this case, *H*_c_ represents the combined hardness directly extracted from the measurement. *H*_f_ and *H*_s_ are the film and substrate hardness, respectively. Then *k* is a constant that depends on the film properties, and *h* represents the total displacement of the indenter, typically normalized to the film thickness. From the equation it is clear that the true value of *H* for the film can be extracted from the fitting when *h* tends to zero. Additionally, it is important to note that although the example is presented for *H*, it is also applicable to *E*.

**Figure 7:**
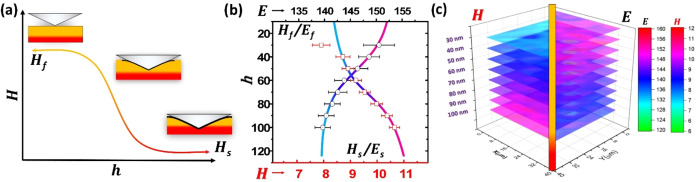
(a) Graphical representation of the Korsunksy method and its typical plot. The different stages of the indentation experiment are shown as insets. (b) Experimental data were extracted from a 100 nm nanolaminated film. The increments in depth are shown in nm, and the values for *H* and *E*, are presented in GPa and marked with the symbols red square and ○, respectively. (c) Topographic representation of a 40 × 40 μm^2^ area, each depth presented as a topographic map, on the left *H* and on the right *E*. Taken with permission from ref 53.

A real case in which nanoindentation and the Korsunsky method are used to study nanolaminates of titanium dioxide (TiO_2_) and aluminum oxides (Al_2_O_3_) is presented in Figure 7b. In the example, the depth profiling and extraction of mechanical properties (*H* and *E*) is performed.^53^ Notice that the films are ∼100 nm in thickness and have been deposited over a Si(001) substrate. Nominally, Si substrates have an *H* = 11–13 GPa and *E* = 125–145 GPa,^54^ which provides a good frame of reference for the *H*_s_ and *E*_s_. Additionally, since the samples’ thickness and roughness are known and confirmed by microscopic techniques, the *h* is also known. Therefore, changes in *H* and *E* from the composite surface toward the substrate can be examined in detail. Moreover, since the indenter can perform a matrix of indentations over a large area of the substrate, in the case of Figure 7c a 10 × 10 matrix of indents in a 40 × 40 μm^2^ area, an in-depth topographic reconstruction is possible. This method enables the four-dimensional (4D) reconstruction of nanomechanical information since, in the case of Figure 7c, a plot with information can be generated, such as (*x, y, z*, *E*-*H)* but potentially many more. Including the elastic recovery (*W*_e_), ratios like the elasticity index (*H*/*E*)^31^ and the *H* ^3^/*E* ^2^, which play an important role in the tribology of materials.^55^ Finally, in the study described here, by using nanoindentation, it was shown that the reduction of laminate thickness resulted in the increment of interfaces and the reduction in the size of the nanocrystalline particles, which directly influenced the mechanical properties of the nanolaminates. Additionally, it proved beneficial by preventing the failure and wear of the film due to mechanical work.

Another example of nanoindentation is the case in which two competing film phases coexist. In general, X-ray diffraction (XRD) or local high-resolution transmission electron microscopy (HR-TEM) experiments should provide an idea of the crystal structure of each phase. However, there are some cases in which the differences are almost negligible, the materials are not polycrystalline enough to provide a meaningful comparison, or the studies are unavailable. Recent advancements in high-speed scanning nanoindentation provide possibilities for a rapid assessment of the spatial difference in mechanical properties between neighboring phases.^17,18^ An example of this is the thermally reconstructed Gadolinium Molybdate Gd_2_(MoO_4_)_3_ (β′-GMO) thin films,^56^ atomic force microscopy (AFM) image shown in Figure 8a.

**Figure 8:**
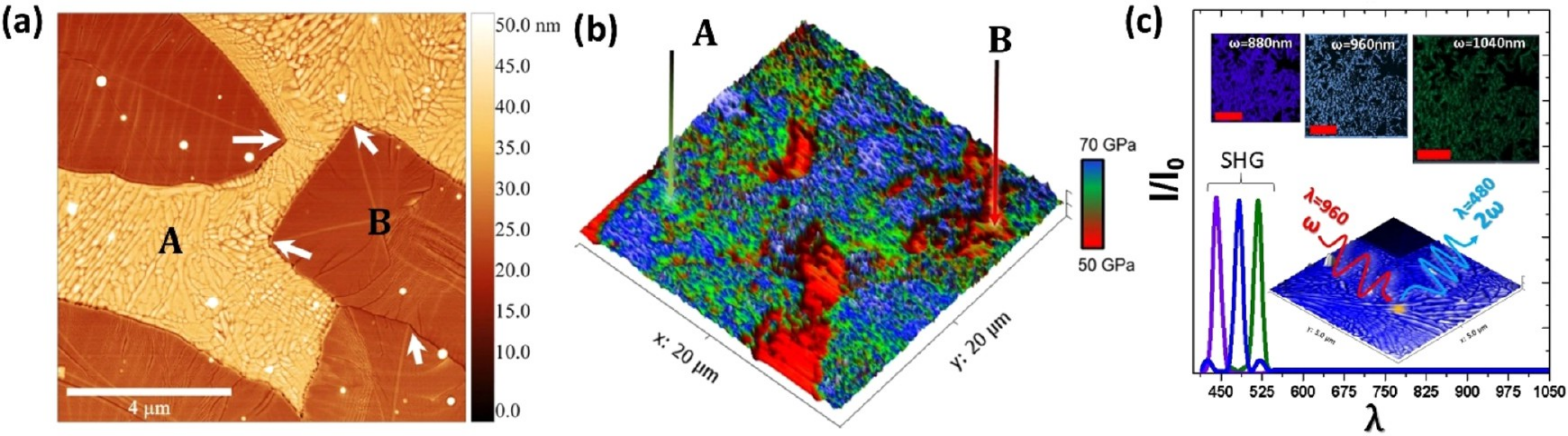
(a) AFM micrograph of the two dominant phases in the (β′-GMO) film (A and B). White arrows, show the sharp boundaries of the two phases. (b) Shows a topographic reconstruction of the *E* values extracted from a *h* = 2–5 nm on a 20 × 20 μm^2^ region of the sample. The high values correspond to the A phase, while the soft bottoms correspond to the B phase. (c) Second harmonic generation (SHG) maps of the sample show that phase A shows a strong SHG signal, which is a characteristic of the ferroelectric phase. Reproduced from ref 56 Available under a CC-BY 4.0 license. Copyright 2017 Springer Nature 2017 Coy. E., Graczyk, P., Yate, L., et al.

GMOs are well-known but still promising. GMO is a full ferroelectric–ferroelastic material with a complex phase diagram,^57^ effective P(001): 2 μC/cm^2^ polarization and coercive stress of 1 MPa.^58^ The complex phase diagram is due to its high oxygen content, a critical aspect of the rare-earth molybdates (Re_2_(MoO_4_)_3_), which is worsened by the costability of several off-stoichiometric phases. Nevertheless, the orthorhombic phase of GMO (β′-GMO), is both ferroelectric and ferroelastic. However, due to the aforementioned plethora of crystalline phases, scarce literature has the aim to stabilize GMO in thin films and much less for β′-GMO.^59–62^ In the study discussed here, the ferroelasticity of the film is an important aspect. Amorphous or paraelectric GMO, possess an *E* = 30–50 GPa, while ferroelastic/ferroelectric β′-GMO has a *E* = 60–70 GPa.^63^ Therefore, mechanical topographical maps of the GMO thin film (40 nm) could allow the identification of both phases. In Figure 8b the topographical maps of the films are shown. The difference between *E* in both phases can be clearly visible, allowing us to identify phase A as β′-GMO. Furthermore, Figure 8c, shows the second harmonic generation (SGH) response of the ferroelectric/ferroelastic phase, confirming the mechanical findings. Finally, in this study, the successful reconstruction of ferroelectric β′-GMO was shown by the hand of nanoindentation, and unique topographical mechanical maps on a 40 nm thin film were presented.

### 2.2 Nanomechanical Property Screening for High-Throughput Combinatorial Studies of Thin Films

Combinatorial thin-film synthesis and high-throughput characterization methods have been developed in the last decades, leading to the discovery and development of a large diversity of new materials and accelerating a broad range of materials research efforts for the determination of composition–structure–processing–property relations.^64–67^ Combinatorial thin-film synthesis relies on the deposition of thin-film libraries with composition gradients across the combinatorial sample libraries by cosputtering (usually magnetron sputtering) from multiple sources at incident angles. The simplest case of combinatorial thin-film deposition is presented in Figure 9a for cosputtering from two sources at an oblique angle. Moreover, the orthogonal temperature gradients can be applied simultaneously during deposition by only partially clamping the substrate to the heated substrate holder that provides sample libraries grown at different temperatures (Figure 9b).^68^ Additionally, combinatorial synthesis of wedge-type multilayer films composed of nanoscale wedge-type layers can be carried out by using computer-controlled movable shutters followed by postdeposition annealing to achieve phase formation.^69^ The large substrate can then be divided into a measurement grid consisting of several discrete arrays of measurement areas (samples) sufficiently separated from one another. A high-throughput high-quality characterization using a comprehensive suite of automated characterization tools is applied to the synthesized libraries in the following step.^70–72^ Combining these results into one data set using specialized software allows rapid characterization of thin-film materials’ structural, physical, and functional properties. Characterization tools used in a combinatorial workflow can be divided into categories considering the properties to be characterized: (i) basic structural (phase composition and microstructure) and compositional (elemental composition, chemical bonding) properties, (ii) functional properties (like electrical conductivity, optical properties, and magnetic properties^73^), and (iii) mechanical properties such as hardness, yield strength, Young’s modulus, and residual stress. Although there are a number of techniques developed to test the mechanical properties of thin films and small-scale materials and structures, nanoindentation is at the frontier in mechanical characterization of thin-film material libraries for mapping hardness and Young’s modulus, first of all, because it is operationally simple, automatized, user-friendly, and fast performing. Since high-throughput mechanical characterization requires fast testing of a large set of combinatorial samples in either parallel or scanning mode, nanoindentation is a perfect tool for characterizing mechanical properties in combinatorial studies. Despite the fact that combinatorial synthesis was initiated in the middle of the previous century,^74^ the first publication involving nanoindentation into combinatorial workflows is dated back to 2002 for the Pd–Pt–Rh trijunction area of the diffusion multiple.^75^ There are two basic approaches for screening materials properties of combinatorial libraries by nanoindentation: (i) scanning nanoindentation of a combinatorial sample with continuous composition grading (Figure 9c) and (ii) sequential Indentation in discrete arrays representing samples (Figure 9d). (i) is rarely used in combinatorial studies simply because there is no grouping by samples that complicates a high-resolution correlation between local mechanical properties and local composition/crystallography or other functional properties. Moreover, multiple indentations per sample point are necessary to statistically determine the average hardness/modulus per composition and determine measurement error. Since the first attempt to integrate nanoindentation into combinatorial workflow, it allowed to elucidate structure/composition–mechanical properties relationships for binary alloys (W–Cu,^76^ Ti–Cu,^77^ Ag–Al,^78^ and Ti–Al alloys^76^), high-entropy alloys,^76^ thin-film metallic glasses,^79–82^ shape-memory thin-film alloys^83,84^ and hard ceramic coatings.^85,86^

**Figure 9:**
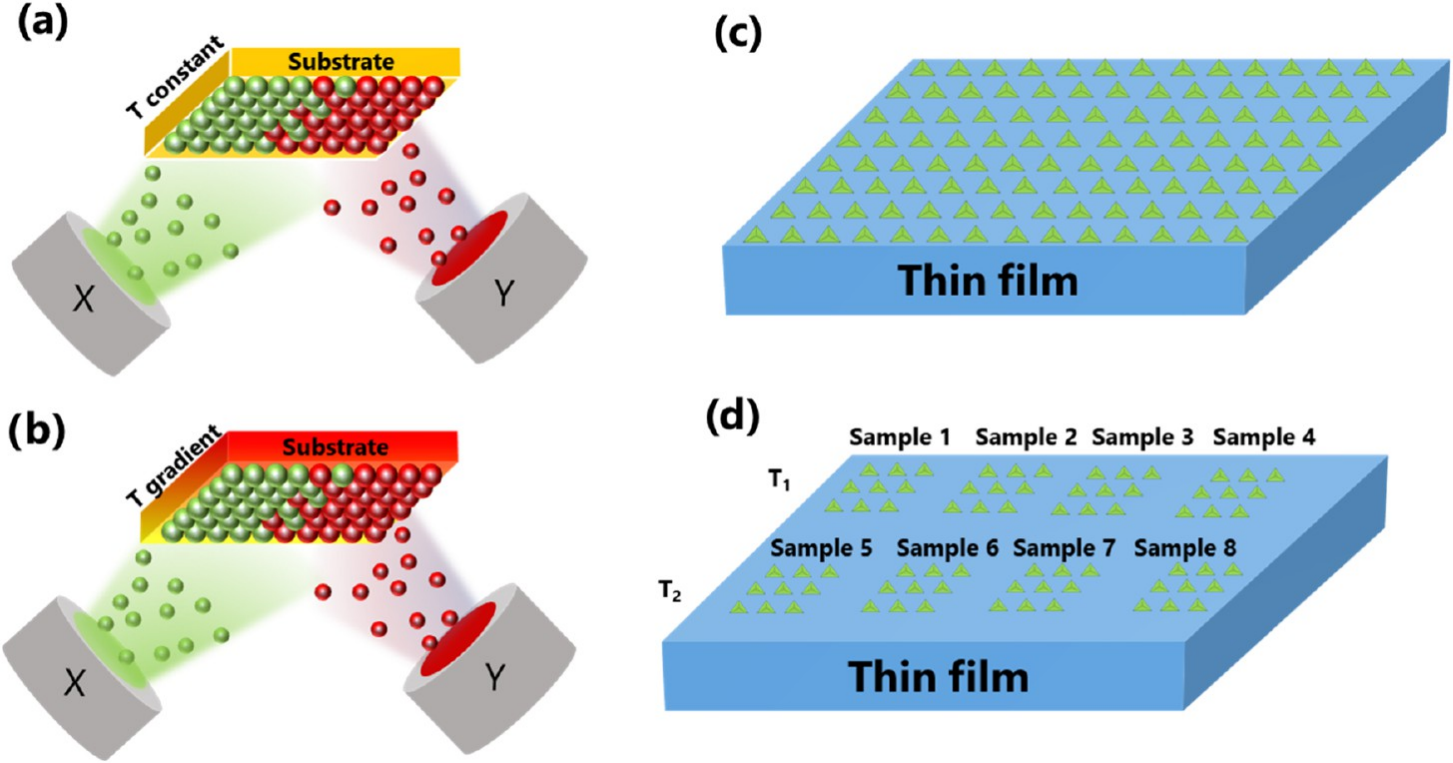
(a) Schematic illustration of combinatorial synthesis of a complete binary system (X and Y denote two different materials) by magnetron cosputtering from two sources on the substrate with constant substrate temperature and (b) with orthogonal gradient of the substrate temperature. (c) High-throughput nanoindentation by scanning nanoindentation of a sample with continuous composition grading and (d) sequential indentation in discrete arrays representing samples of different compositions grown at different substrate temperatures (*T*_1_ < *T*_2_).

An example of hardness mapping by nanoindentation correlated with EDS-derived composition is shown in Figure 10a for Ti–Al thin-film library grown by magnetron cosputtering after applying corrections to Oliver and Pharr method to account for pile-up and the elastic modulus mismatch between the film and substrate with increasing indentation depth^87^ (Figure 10b). Although the measured hardness of thin films is higher than that of bulk alloys due to the indentation size effect and differences in microstructure, the general trends obtained from the hardness mapping for thin films closely track the properties of bulk alloys. This similarity of the trends in hardness suggests that the combinatorial approach is feasible for the rapid identification of compositions that might merit development as bulk structural materials. Another study of Thienhaus et al.^88^ on Al–Ni thin films illustrates that the high-resolution screening of mechanical properties yields data with clear nonlinearities (maxima, minima, inflection points) on which the determination of areas of interest in materials libraries can be based. They reveal that it is difficult to detect correlations between the detected local minima/maxima and inflection points of the hardness and elastic modulus, with possible phases determined by XRD (Figure 10b,c). This is because the error of the measurements is often in the range of the individual local minima/maxima. However, the decrease in hardness and modulus around Ni_76_Al_24_ coincides with the phase boundary identified by XRD (Figure 10c). Meanwhile, the increase of *H* and *E* with Ni content is assigned to a build-up of inner stresses that lead to film delamination in the Ni range between 43 and 51 atom %. Zarnetta et al.^83^ revealed the increase in Young’s moduli and increased scatter in the data for compositions deviating from Ti content of 50 atom % in Ti_*x*_Ni_90–*x*_Cu_10_ (37 atom % < *x* < 67 atom %) shape-memory alloy thin films. This originates from significantly different load–displacement responses (Figure 10i) of the phases present at the location of the tip revealed by high-angle annular dark field (HAADF) transmission electron microscopy (TEM), determining the indentation results (Figure 10e–i). This typically requires separating the indentation responses into groups and analyzing them individually for the estimation of *H* and *E* of the different phases.

**Figure 10:**
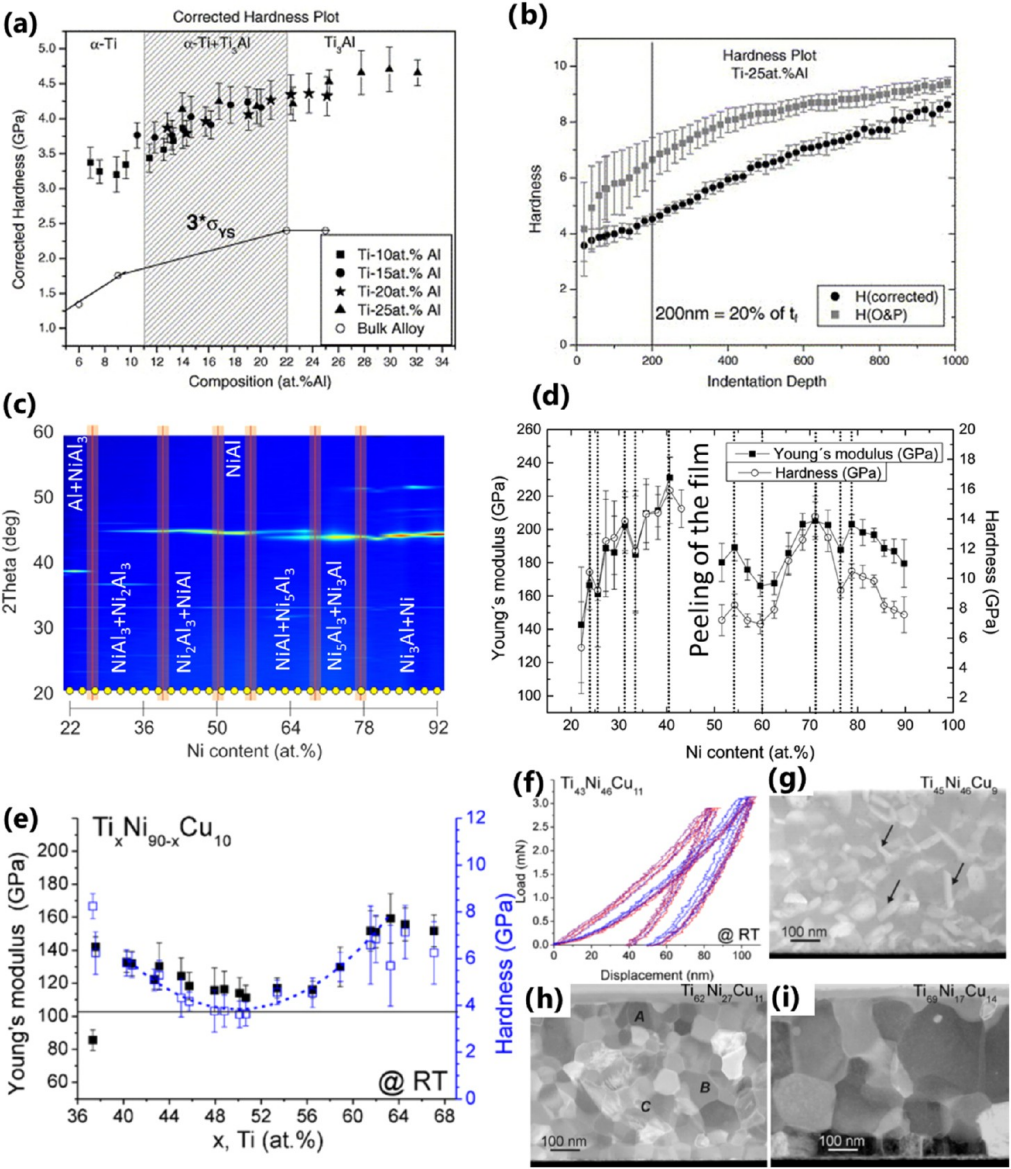
(a) Hardness as a function of composition for Ti–Al thin-film libraries showing a trend similar to the strength of bulk alloys (applying Tabor’s criterion, *H* = 3σ_*y*_). The shaded region marks the two-phase region of α-Ti + Ti_3_Al. (b) A comparison of the typical hardness vs indentation depth plots analyzed using the Oliver and Pharr method (plotted in gray, H (O&P)) with the plots analyzed using the method developed by Han and co-workers (plotted in black, H (corrected)) to account for pile-up and the elastic modulus mismatch between the film and substrate with increasing indentation depth.^87^ (c) Visualization of XRD patterns taken along the compositional gradient from Ni_22_Al_78_ to Ni_92_ Al_8_ (blue, low; red, high peak intensity). The broad red lines indicate the phase boundaries of the identified phase regions. Reproduced from ref 88 Copyright 2014 American Chemical Society. (d) Nanoindentation hardness and Young’s modulus for Ni–Al thin films with composition from Ni_22_Al_78_ to Ni_92_Al_8_. Local maxima and minima are marked with dotted lines. Reproduced from ref 88 Copyright 2014 American Chemical Society. (e) Composition dependencies of *H* and *E* for Ti_*x*_Ni_90–*x*_Cu_10_ shape-memory thin-film samples.^83^ (f) Sets of 10 load–displacement curves with bimodal distributions for the Ti_43_Ni_46_Cu_11_ thin film with a multiphase microstructure. High-angle annular dark field (HAADF) TEM images of Ni-rich (g) Ti_45_Ni_46_Cu_9_ and Ti-rich (h) Ti_62_Ni_27_Cu11 and (i) Ti_69_Ni_17_Cu_14_ thin films annealed at 500 °C. Arrows in (g) indicate Ti­(Ni,Cu)_2_-rich precipitates. Letters A–C in (h) indicate different phases with the following compositions: Ti_60_Ni_19_Cu_21_, Ti_63.5_Ni_33_Cu_3.5_, and Ti_52_Ni_32_Cu_16_. Taken with permission from ref 83.

Ti–Cu thin films with different composition ratios were created using a combinatorial synthesis approach while mechanical properties were mapped by nanoindentation to elucidate the mechanism underlying yield strength, i.e., the onset of dislocation motion, employing the Neural Network potential combined with molecular dynamic simulations.^77^ Moreover, owing to its high-throughput nature, it is most valuable for high-entropy alloy fields, allowing mechanical characterization of multicomponent alloys with large composition intervals. Among all, this allowed to reveal that Cr seems to be the most potent strengthening element due to its highest strengthening parameter in solid solution strengthening in Cantor alloys (CrMnFeCoNi equiatomic alloy)^86^ as well as to screen the phase formation trends in AlCrFeNiTi compositionally complex alloys, helping in reveling strengthening mechanisms such as precipitation hardening and dispersion strengthening.^89^ Moreover, the high-throughput nanoindentation study of Cu–Zr thin-film libraries has provided quantitative data for establishing new structural models and a fast and direct way to search for new glass-forming alloys, leading to guidance for the development of ductile bulk metallic glasses.^80^

A combinatorial adhesion study employing nanoindentation by conical diamond tip producing well-defined delamination-blisters, Figure 11, was performed to calculate and map the interfacial fracture energy for AlCuAu and AuAgPd alloy thin films covered with Al_2_O_3_.^90^ This allowed the reveal of areas of optimum adhesion layer composition, demonstrating the best adhesion properties. Overall, higher adhesion energy values were measured for AlCuAu, Figure 11c, compared to those of the ternary noble metal layers with the highest adhesion in a two-phase, Cu-rich composition region. Meanwhile, the lowest adhesion corresponds to intermetallic regions. Moreover, the failing interface changes with Al content (Figure 11d): in the Al-rich area, delamination occurs between the adhesion layer and Al_2_O_3_, while at higher Au content the delaminating interface is located between the sapphire substrate and the adhesion layer.

**Figure 11:**
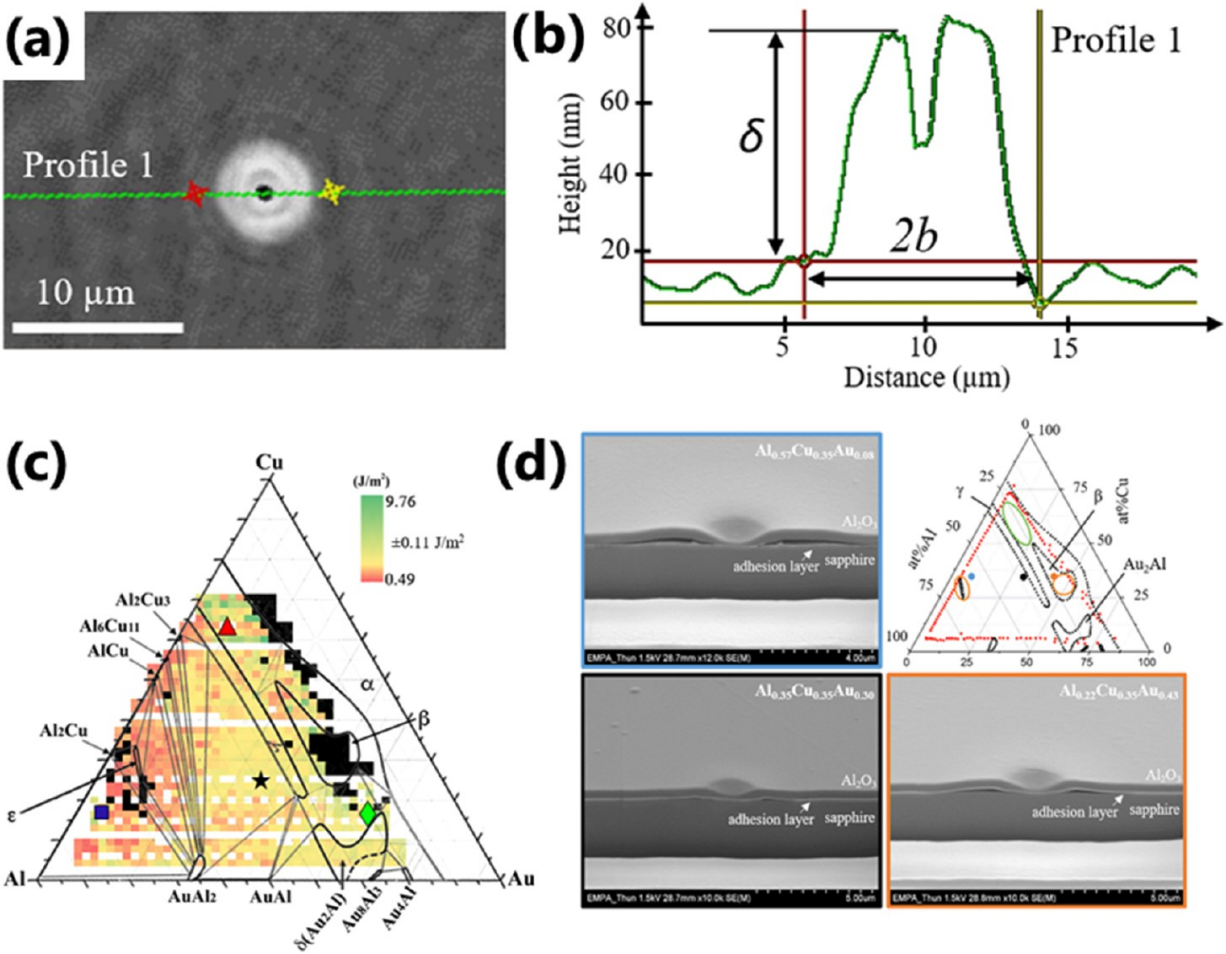
(a) Digital holographic microscopy image and (b) corresponding height profile of a local delamination induced by nanoindentation using a conical diamond tip (2 μm radius, 120° opening angle) used for the determination of the blister diameter and height. (c) Adhesion map for the ternary AlCuAu alloy thin film with the ternary phase diagram as an overlay. (d) Focused ion beam milled scanning electron microscopy (FIB-SEM) cross-section images of indentation blisters for Al_0.57_Cu_0.35_Au_0.08_, Al_0.35_Cu_0.35_Au_0.30_, and Al_0.22_Cu_0.35_Au_0.43_ and their relative position on the wafer.^90^ Reproduced from ref 90 Available under a CC-BY 4.0 license. Copyright 2020 Schoeppner, Ferguson, Pethö et al.

The examples presented above and recent works have determined the challenges of high-throughput nanoindentation. It is well established that care must be taken to avoid errors in such measurements, including indentation depth and indentation spacing, pileups, and impact of microstructural features—all previously reviewed by Hintsala and collegues.^17^ When performing nanoindentation on large samples (such as 4 in. wafers typically used in combinatorial studies), the effective mechanical compliance of the components representing the load frame of the instrument must be largely independent of indent location. It was stated in early works that even for the binary alloy case, nanoindentation requires too long measurement times for the rapid identification of areas of interest in materials libraries.^88^ However, recent technological developments in nanoindentation devices allow high-speed nanoindentation to be performed as fast as up to 6 indents/second, representing at least 2 orders of magnitude improvement over standard quasi-static tests.^17^ As thousands of indents must be performed per a combinatorial sample, the calibration accuracy for the diamond tip shape area becomes of exceptional importance.^18^ Microstructure and mechanical properties are intimately linked, so they should not be considered separately. This has been shown above for shape-memory alloys due to the complexity of their phase composition. Moreover, abnormal tilted grain growth following the empirical cosine and tangent rules is a very common phenomenon in an oblique angle sputter deposition of thin-film combinatorial libraries. These tilted grains are difficult to detect using conventional XRD in Bragg–Brentano geometry. This must be accounted for when analyzing nanoindentation data in conjunction with XRD. Apart from the mapping of *H*, *E*, *H*/*E* and *H* ^3^/*E* ^2^ ratios, mapping of *W*_e_ that can be calculated directly from nanoindentation force–displacement curves taken under conditions of low indentation-induced strain can be a simple and relatively high-throughput means of mapping superelastic regions of thin-film compositional libraries.^16^

Nanoindentation as a rapid detection tool is significant because it complements other techniques used in high-throughput characterization loops and provides quantitative values for various mechanical properties. It also has the advantages of much higher composition resolution and not necessitating the fabrication of additional special libraries as compared to yield strength measurements (micropillar fabrication) or fracture toughness measurements (cantilever fabrication). Combining nanoindentation and other characterization techniques allows us to allocate “sweet spots” of the best performance and optimize thin-film properties for engineering applications. Moreover, as more data are accumulated, databases of hardening/strengthening mechanisms can be built, becoming a valuable tool for thin-film material design and providing guidance for elemental selection for new complex alloy systems.^91^ Additionally, the temperature- and environment-dependent phase evolution of complex materials can be studied in an accelerated way using combinatorial processing platforms coupled with nanoindentation.

### 2.3 *In Situ* and *In Operando* Nanoindentation Techniques

There is a large number of *in situ* nanoindentation techniques that can be performed in (I) analytical instruments, like TEM^92^ or SEM,^93^ XRD including beamlines using synchrotron irradiation,^19,94^ or (II) under various environmental conditions, like low or high temperatures, applied currents, and field, elevated humidities^95^ and under different electrochemical conditions.^96^ The first group of *in situ* methods was utilized to understand different materials’ mechanical properties and deformation mechanisms thoroughly.^97^ The second group of techniques, usually called *in operando*, aims to understand the mechanical properties of materials under conditions that closely mimic their operational environment. Combining these two approaches, for instance, high-temperature Indentation in TEM or SEM, allows the study of the mechanical behavior of different high-temperature alloys and ceramics. Moreover, nanoindentation combined with electrical nanoprobing inside the SEM can yield microscale insights into how mechanical pressure and deformation affect the electrical properties of different materials and devices.^98^

For a comprehensive and in-depth discussion on the topic of *in situ* nanoindentation, readers are encouraged to consult the extensive reviews and research articles available in the literature (Table 1). Notably, the works by Zhong et al.^99^ and Minor^21^ provide detailed analyses and a broader context for *in situ* nanoindentation in TEM, offering a thorough understanding that exceeds the scope of this article. Further valuable insights can be found in the review by Nili and co-workers,^100^ which systematically addresses the current advancements and challenges in integrating nanoindentation with real-time electron imaging, high-temperature measurements, electrical characterization, and a combination of these.

Notably, mechanical properties of some materials can be characterized exclusively using *in situ* methods, such as nanomaterials (nanoparticles,^101^ nanowires, 2D ultrathin films,^102^ nanotubes,^103,104^ nanopillars,^105^ single biodegradable fibers^106^) and living cells.^107–110^ This usually necessitates the utilization of AFM-integrated indentation-based methodologies.^26,27^ AFM-based nanoindentation techniques differ from the conventional nanoindentation test in terms of contact mechanics consideration for the estimation of mechanical properties from the force–displacement curves. Mechanical properties are deduced from nanoindentation experiments following the elastic model of contact for contacting spherical surfaces developed by Hertz^111^ and extended to a range of indenter geometries by Sneddon.^9^ This method was further refined by Oliver and Pharr^14^ to account for the changing contact area at different points in the unloading curve. This approach is applicable when a rigid, sharp indenter with well-defined geometry (typically Berkovich, Vickers, or spherical diamond tip) is used producing a large contact area, and penetration depth can reach hundreds of nanometers to microns. In contrast, AFM techniques use a cantilever with a nanoscale tip, often conical or pyramidal, sometimes spherical, producing a relatively low contact due to ultralow load force, nN to μN range. However, at such conditions the pure Hertzian model does not consider surface energies and related adhesion forces, roughness, and capillary forces, which become significant and may even dominate the overall force behavior at the nanoscale.^112^ To account for this nanoscale effect, Johnson–Kendall–Roberts^113^ (JKR) and Derjaguin–Muller–Toporov^114^ (DMT) models have been developed paving the way for a reliable measurement of mechanical properties of nanoscale materials using AFM-based nanoindentation.

The quest for sustainable development has sparked the development of materials for energy storage and the generation of low-carbon-emission energy. Mechanics and electrochemistry are coupled in such energy technologies as batteries and fuel cells.^115^ This is first because the electrochemical reaction between the host material and guest species (H_2_ or Li) induces mechanical degradation or embrittlement, which can cause early performance degradation and device failure. However, understanding the intimate coupling between mechanics and electrochemistry is far from complete. This has led to the development of *operando* nanoindentation techniques, allowing to probe the chemomechanical behaviors of electrodes in Li-ion batteries^115^ and hydrogen-induced mechanical degradation of materials comprising a fuel cell.^15,115^

#### 2.3.1 *In Situ* Nanoindentation during Electrochemical Hydrogen Charging

Starting in 2006, nanoindentation paved the way for the study of hydrogen-material interactions.^116^ The interaction between hydrogen and structural materials, particularly stainless steel, has led to various industrial challenges, and hydrogen embrittlement (HE) has been a great issue since its first discovery in 1875. Understanding the HE is critical for ensuring the safety and reliability of structures exposed to hydrogen, such as pipelines, storage tanks, and fuel cells. Although many authors have thoroughly studied both hydrogen embrittlement and hydrogen-induced fracture phenomena, this research was done mainly macroscopically.^117–119^ However, material failure mechanisms begin at the atomic scale, initiated by hydrogen absorption and its subsequent interaction with defects or trap binding sites.^15^ These defects can range from vacancies and interstitial atoms to dislocations, grain or phase boundaries, and precipitates. In this case, nanoindentation, due to its ability to probe small material volumes, would serve as an effective technique to independently examine those processes.

Barnoush and Vehoff were able to perform the first nanoindentation tests with *in situ* charging, introducing the pioneer electrochemical cell implemented in a Hysitron Triboscope in conjunction with a Digital Instruments (DI) Nanoscope II.^116^ A three-electrode nanoindenter setup was developed, as shown schematically in Figure 12a. Pt wire was used as the counter electrode, and Ag/AgCl was used as the reference electrode. Nanoindentation tests were provided inside the electrochemical cell made from TeflonTM while the sample was covered with approximately 2 mm of a 0.05 M Na_2_SO_4_ electrolyte prepared from an analytical grade Na_2_SO_4_ and double distilled water. This system was placed in a protective nitrogen and helium atmosphere to eliminate the oxygen effect during the electrochemical reaction. The mean tip radius (500 nm) was measured by making elastic indentations and fitting the loading part with a Hertzian contact model.^123,124^ This setup provided reproducible results since scattering due to variations of electrochemical and surface conditions was removed. The sample was kept at a cathodic potential of −1000 mV during the filling of the electrochemical cell with the electrolyte through a MicroFillTM pipette. Experiments were implemented on a (111) Ni surface. This setup was used by different research groups for several years.^125–128^

**Figure 12:**
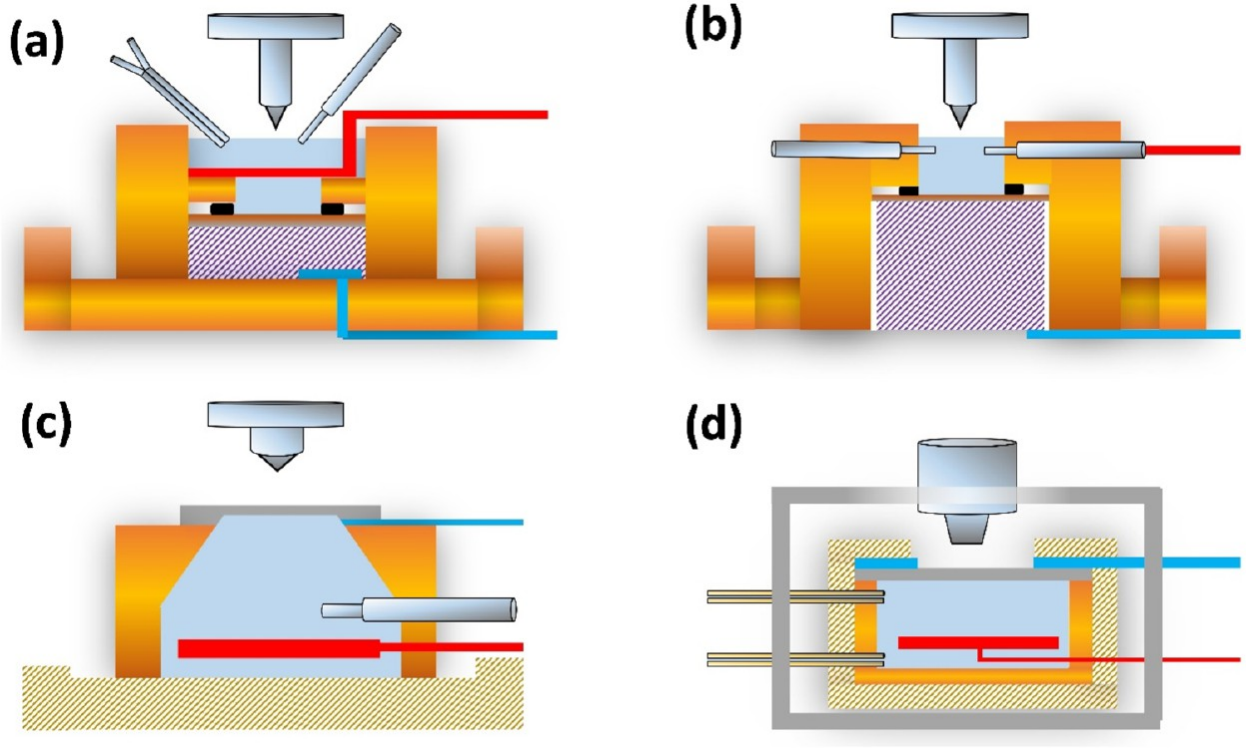
Schematic representation of different *in situ* electrochemical setups for nanoindentation; (a) first top-charging approach by Barnoush et al.;^116^ (b) adapted top-charging for battery materials by Epler et al.;^120^ (c) back-side charging developed by Duarte et al.;^15^ and (d) *in situ* setup for application in SEM with back-side charging by Kim et al.^121^ Image based and redrawn from ref 122.

Another electrochemical setup developed for the G200 nanoindenter (KLA Tencor, former Keysight Aglient) was mentioned in the thesis of Epler^120^ and is presented in Figure 12b. This setup was applied to study the degradation of battery electrode materials during repeated charging cycles and kept the concept of front-side charging.

The sample studied was mounted from below for both of these front-side approaches. So, there is a huge disadvantage of the missing movement of the stage in the *z*-direction for both of these strategies. Kim et al.^121^ implemented an electrochemical setup into a SEM for continuous imaging at charging and indentation. The main challenge for implementation in an SEM was the necessity for maintaining a good vacuum level. A back-side charging approach was performed to achieve this goal, as presented in Figure 12d. For this setup, the electrolyte was filled into a closed reservoir on the sample’s bottom, and measurements were provided on the top. The absorbed hydrogen must diffuse through the sample to the testing surface. Therefore, the sample thickness must be adjusted to the diffusibility of hydrogen for the material tested. Unfortunately, this modification reduces stiffness, affecting the stiffness-related nanoindentation results. Nevertheless, different research groups still apply similar back-side charging approaches (Figure 12c).

Recently, Duarte et al.^15^ provided a comparison of the advantages and disadvantages of the front and back-side charging setups for electrochemical nanoindentation. Their experiments have shown a decrease in hardness and elastic modulus values at the front-side charging, while an increase in hardness and negligible change in the elastic modulus was defined at the back-side charging approach.^15^ Summarizing all the results obtained, the authors highlighted both methods’ advantages and disadvantages.

An undoubted advantage of front-side charging is the fast charging of the testing surface, which reduces concentration gradients in the volume tested. While the main disadvantage is referred to surface evolution/damage at charging that shortens the testing time and limits the range of possible charging electrolytes used. Besides, the sample should be polished between tests, which may disturb the surface structure.

The advantages of the back-side charging include very high reproducibility and repeatability since the testing surface of a sample is not affected or damaged by the charging. The method can ensure time-dependent measurements by taking hydrogen absorption and diffusion through the material into account. The primary drawbacks of this method are the extended time required for hydrogen to reach the testing site in alloys with low hydrogen diffusivity, such as FCC metals, and the risk of hydrogen desorption if an oxygen-free environment is not maintained.

With various experimental setups, classical nanoindentation tests can be used to investigate the sensitivities of different materials to hydrogen charging. The primal experiments of Barnoush and Vehoff^116^ were focused on a nickel (111) single crystal. The analysis centered on the “pop-in” phenomenon, which occurs at the transition from fully elastic to elastic-plastic deformation, visible as a sudden displacement burst in the load–displacement curve. The first few nanometers of this curve, featuring a pronounced pop-in, are illustrated in Figure 13a. According to the literature, the pop-in is associated with homogeneous dislocation nucleation (HDN) under the indenter tip in a material that is nearly free of dislocations.^129^ This process is schematically represented in Figure 13b. Such a quasi-dislocation-free condition is typically observed in coarse-grained materials with low intrinsic defect densities (e.g., dislocations, precipitates, and inclusions). Under these conditions, the pop-in load correlates with the radius of the indenter tip and the material’s maximum shear stress, as described by

10 $$\tau_{\max}=0.31{\left(\frac{6E_{r}^{2}}{\pi^{3}R^{2}}P_{\mathrm{pop}‐\mathrm{in}}\right)}^{1/3}$$

where *E*_*r*_ is a reduced modulus, *P*_pop‑up_ is a pop-in load.

**Figure 13:**
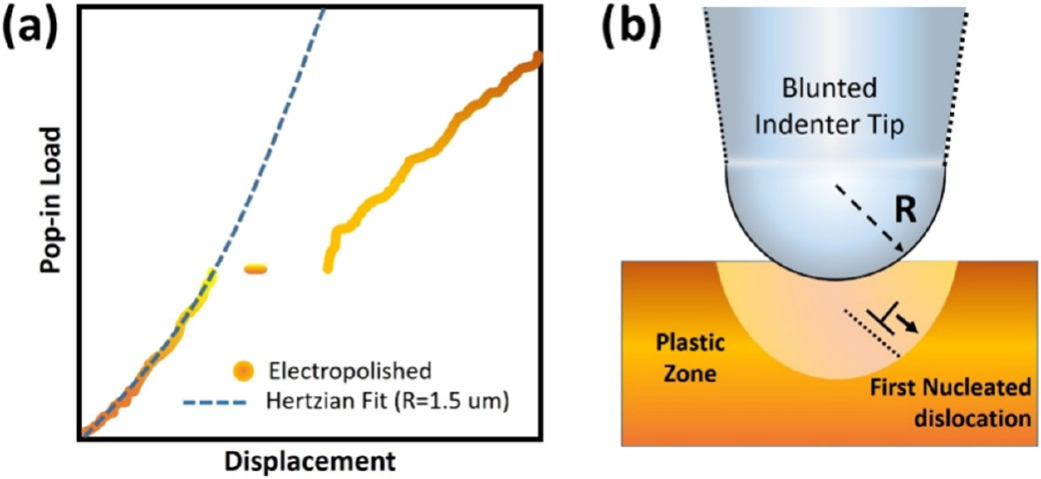
Overview of pop-in formation; (a) load–displacement curve with pronounced initial pop-in for an electropolished nickel-based alloy with a conical tip (specified radius 1.5 μm) and (b) schematic illustration of blunted indenter tip with radius *R* and first nucleated dislocation in a quasi-dislocation-free material that subsequently developed a plastic zone under the indenter tip.^122^ The dislocation shown in (b) occurs inside a quasi-dislocation-free material creating a plastic zone and the arrow points on the direction of the dislocation movement toward the boundary between the quasi-dislocation-free material and plastic zone. Inspired and redrawn from ref 122.

The reduction in the pop-in load due to hydrogen charging was first attributed by Barnoush et. al^116,130^ to hydrogen weakening the lattice cohesion, facilitating dislocation nucleation beneath the indenter tip. Further studies allowed to development a combined HEDE (Hydrogen-Enhanced Decohesion) and HELP (Hydrogen-Enhanced Localized Plasticity) model,^130–132^ which suggests that hydrogen weakens interatomic bonds by reducing the shear modulus or stacking fault energy.

In 2012, the scope of electrochemical nanoindentation studies was expanded to include measurements of hydrogen-induced hardness increases in austenitic steel.^127^ Beyond pop-in analysis, this hydrogen-induced hardness increase became a standard feature of electrochemical nanoindentation studies. The technique has since been applied to a wide range of material classes, including nickel-based alloys,^133^ iron,^134^ steels,^135,136^ and high-entropy alloys (HEAs).^137–139^ The observed hardness increases are explained by reduced dislocation mobility, higher lattice friction between dislocations and hydrogen, and solid solution hardening effects.

#### 2.3.2 *Operando* Nanoindentation of Li-ion Battery Electrodes

Mechanical stresses induced by electrochemical reactions regulate mass transport, charge transfer, and interfacial reactions that strongly impact the potential and capacity of electrochemical systems.^140^ This consequently limits the implementation of high-capacity electrodes in batteries and comprises the performance of current technologies. Electrochemistry and mechanics of both the cathode and anode have been studied separately in recent years. However, the link between mechanics and electrochemistry is far from understanding because the mechanical response of materials at chemical equilibrium may differ from that under concurrent mechanical and chemical load.^141,142^ Since oxygen and moisture can cause numerous side reactions, mechanical tests must be performed under environmental control. de Vasconcelos and co-workers proposed an experimental platform of *operando* nanoindentation that probes the dynamic mechanical behaviors of electrodes during real-time electrochemical reactions.^115^ The nanoindenter, a home-developed fluid cell, and an electrochemical station were integrated into an argon-filled glovebox, Figure 14a. Such a setup allowed us to evaluate the influence of the argon environment, electrolyte solution, structural degradations, and volumetric change of electrodes upon Li insertion, and the effects of the solid electrolyte interface and the substrate. Control experiments validating the custom work conditions were performed by comparing the elastic modulus of fused silica and amorphous silicon measured in the air versus argon environment, in the standard holder versus the dry fluid cell, and on dry material versus wet sample submerged in the electrolyte-filled fluid cell. The closer values confirm that the effects of the dielectric constant of Ar on the capacitance gauge of nanoindentation, the nonstandard sample holder, and the buoyance and surface tension of the liquid electrolyte are negligible, Figure 14b. In order to eliminate the effect of electrochemical drift caused by Li insertion on the estimated contact depth and consequently on the area of contact, the displacement correction procedure based on the thermal drift correction was applied. This correction is based on keeping the load constant during the thermal drift period, while measuring the displacement of the tip. The rate of change of the tip displacement with time gives the thermal drift rate. Multiplying this value with the elapsed time and further subtracting this result from the indentation depth provide the corrected tip displacement. The correction is effective exclusively when the drift is approximately linear for the entire time of the test. Analogously, the electrochemical drift can be attributed to the electrochemically induced volume change due to Li insertion or extraction. However, this is the case only if the residual creep, thermal fluctuations, and the change of the mechanical properties of the sample during the indentation test are negligible. The electrochemical drift was investigated by designing experiments with minimized environmental fluctuations (long stabilization time, no air flow, vibration isolation, and temperature-controlled environment) and sufficient dwell time. In this way, the electrochemical drift rate of Si was measured during the titration experiments under a relatively slow cycle and a relatively fast charging case, respectively. Results clearly showed that the drift rate (volume change) is practically zero under the open circuit condition when zero current is provided, negative for a negative current (lithiation), and positive for a positive current (delithiation). This approach provided good agreement between the experimental and theoretical predictions of the rate-of-change in the film thickness with respect to the charging rate. The proposed correction procedure is sufficient to account separately or together for thermal and electrochemical drift. The elastic modulus and hardness of Si in the course of Li reactions are measured using the continuous stiffness measurement (CSM) method. The elastic modulus and hardness steadily decrease with Li concentration, which is well within the range reported by *ex situ* measurements (Figure 14d,e). Moreover, the effect of the solid electrolyte interface (SEI) is mitigated because the indenter displacement is this experiment is insensitive to the compliant SEI layer whose modulus is below 10 MPa.^143^ This comparison of *ex situ* and *in operando* measurements also shows that the variation of the experimental procedures (different means to suppress oxidation by covering the sample with inert oil or transferring in inert gas flow) can introduce unnatural transitions in the mechanical properties during lithiation. This usually makes data interpretation difficult and can complicate the discovery of important phenomena. Another benefit of *operando* testing in comparison to wafer curvature is that *operando* indentation allows the measurement of modulus and hardness of electrodes independently from their viscous behaviors because the strain rate and the electrochemical charging rate are separate: the strain rate is provided by the load cell while the electrochemical station controls the charging rate.

**Figure 14:**
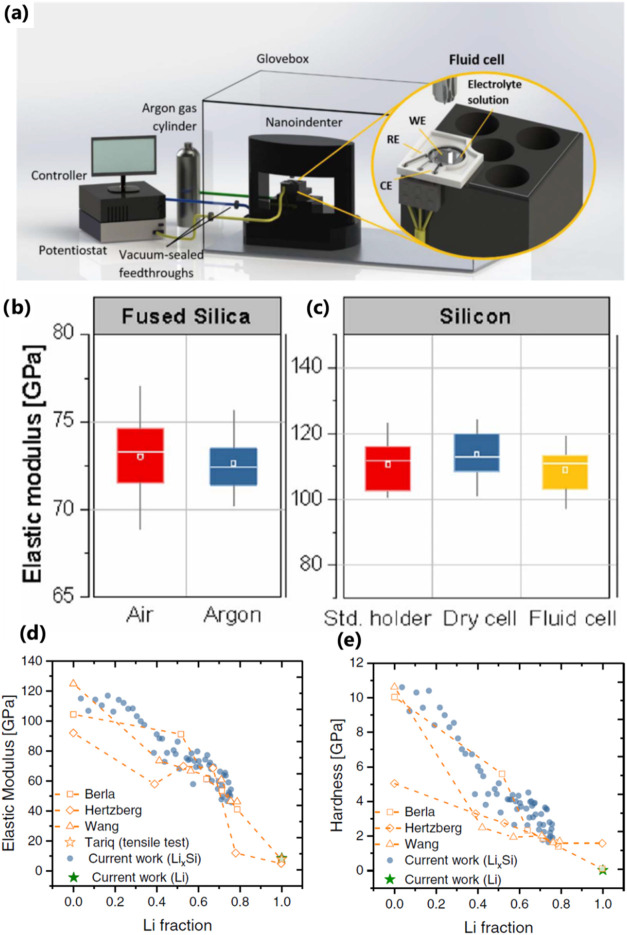
*Operando* nanoindentation of Li-ion battery electrodes. (a) The experimental platform comprises a nanoindenter placed in an argon-filled glow box, a home-built liquid cell, and an electrochemical station. The sample (amorphous Si or Li) serves as the working electrode (WE), while Li metal ribbons are used as the reference (RE) and counter (CE) electrodes. (b) Control experiments performed on (b) fused silica and (c) Si thin film show that indentation tests are not affected by the argon atmosphere, customized holder (dry cell), or the liquid electrolyte environment (fluid cell). (d) Hardness and (e) elastic modulus of Si were measured as a function of Li concentration under various charging states. (d, e) Comparison with the literature results for *ex situ* experiments reported by Wang et al., Hertzberg et al., Berla et al., and Tariq et al. Reproduced from ref 115 Available under a CC-BY 4.0 license. Copyright 2017 de Vasconcelos, Xu, and Zhaoal.

The latter method also allowed the measurement of the mechanical behavior of individual phases in a cathode composite electrode based on lithium nickel manganese cobalt oxides (LiNi_0.5_M_0.3_Co_0.2_O_2_, NMC) in dry and wet conditions. This allowed to probe the influence of electrolyte soaking on the elastic modulus, hardness, and volume change of the conductive matrix with different degrees of porosity. Individual mechanical properties of the poly­(vinylidene fluoride) (PVDF) binder, PVDF mixed with carbon black (CB), and NMC particles in the 1 M LiPF_6_–PC liquid electrolyte as well as NMC pellets are measured by means of *in* *operando* nanoindentation (Figure 15). In the wet conditions, both the elastic modulus and the hardness of the binder drop by about 50% (blue bar, Figure 15b,c). This was assigned to the binder expansion by solvent uptake, which facilitates sliding and disentanglement of the polymer chains. Moreover, the mechanical properties of PVDF and CB/PVDF are relatively the same in the wet state, indicating that the CB loses most of its role in the mechanical response of the composite. The modulus of the dry CB/PVDF composite (2.5–2 GPa, Figure 15d) is about four times that of pure PVDF (0.59 GPa, Figure 15b), while both hardness and modulus decrease significantly with porosity (Figure 15d,e). Figure 15f exhibits the hardness, elastic modulus, and fracture toughness of NMC sintered pellets and secondary particles in dry (red box) and wet (blue box) conditions. The mechanical properties of the agglomerated secondary particles are generally lower than those of sintered pellets, irrespective of the environment caused by weak interfaces between the primary particles in the NMC agglomerate. Despite the mechanical properties of sintered particles remaining relatively unchanged in the electrolyte, the average fracture toughness of the secondary particles is improved from 0.104 MPa m^1/2^ in the dry condition to 0.212 MPa m^1/2^ in the liquid environment. These results suggest that liquid penetration significantly impacts the crack propagating along the interface of primary particles.

**Figure 15:**
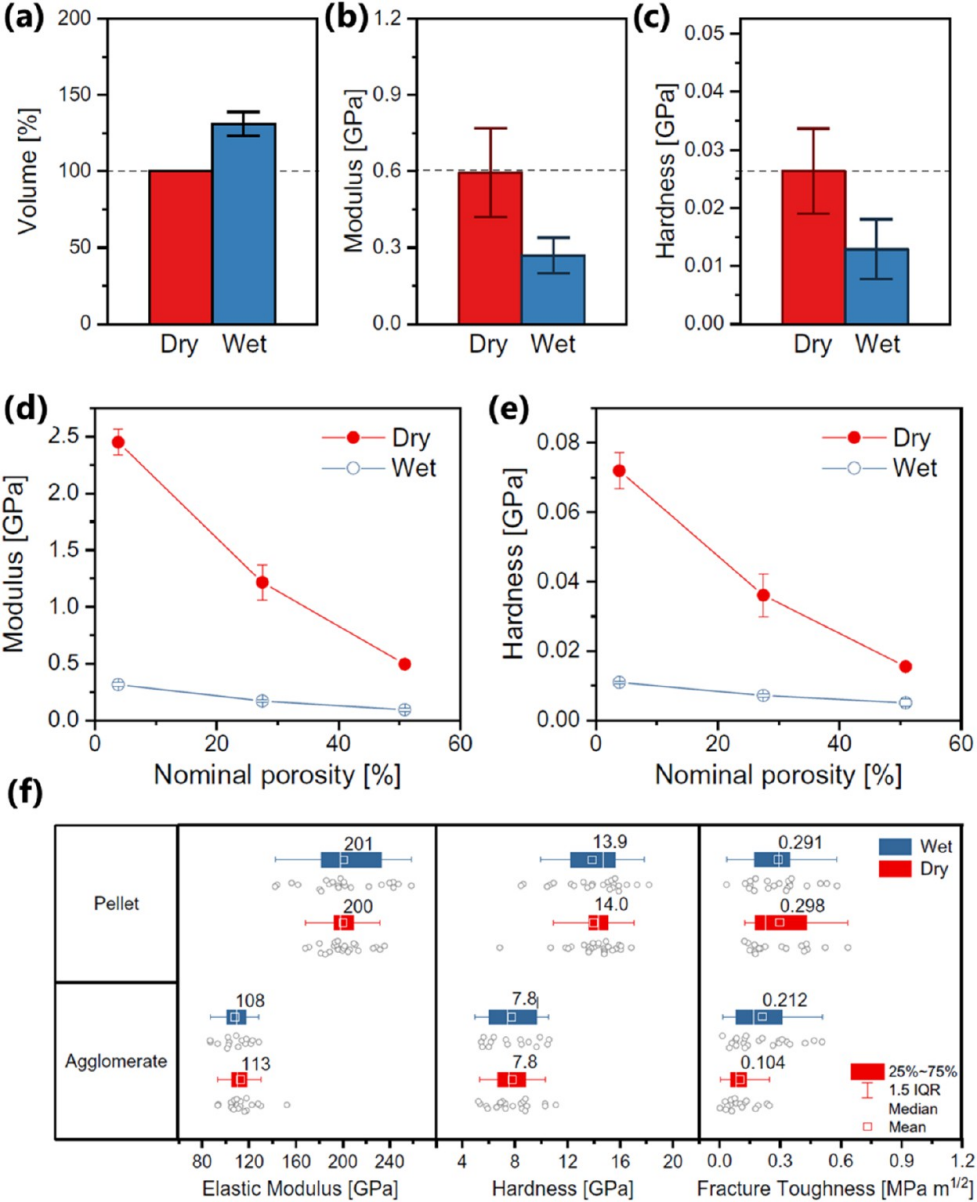
Comparisons of (a) volume, (b) elastic modulus, and (c) hardness of the PVDF binder under dry and wet conditions. (d) Elastic modulus and (e) hardness of the CB/PVDF conductive matrix as a function of the nominal porosity. Dry measurements are shown in red, and wet measurements are shown in blue. The error bars represent the standard deviation. (f) Elastic modulus, hardness, and fracture toughness of the NMC agglomerate and sintered pellet in dry (red box) and wet (blue box) environment. The gray circles represent individual indentations, the box indicates the 25–75% range, the white line is the median, and the average values are shown in the labels and indicated by the white squares.^144^ Taken with permission from ref 144, Copyright 2018 Springer Nature.

### 2.4 Perspective Applications

#### 2.4.1 Mechanical-induced Electronic Effects (Flexoelectricity and Metamagnetism)

Flexoelectricity, the generation of electric polarization in response to a strain gradient, has gained significant attention due to its potential applications in sensors, energy harvesting, and tunable electronic devices. Unlike piezoelectricity, which requires a noncentrosymmetric crystal structure, flexoelectricity is a universal property of all dielectric materials, making it particularly relevant for nanoscale systems where strain gradients are naturally large.^145^ Given the fundamental link between mechanical deformation and polarization, nanoindentation has emerged as a crucial tool for characterizing material flexoelectric behavior.^146^

Nanoindentation provides a direct method for inducing localized strain gradients, allowing researchers to quantify flexoelectric coefficients by measuring the generated electrical response under controlled mechanical loading. This technique has been particularly useful in studying thin films and nanostructures, where strain gradients are more pronounced and flexoelectric effects become significant.^145^ However, its application has also been extended to free-standing cantilever beams of potentially nanometric dimensions, with the help of current nanoindentation instrumentation and frequency-dependent measurements (Figure 16a).^147^ By integrating nanoindentation with *in situ* electrical measurements, researchers have been able to probe the coupling between mechanical stress and electric polarization, shedding light on the fundamental mechanisms governing flexoelectricity at the nanoscale.^148^

Furthermore, nanoindentation can play a critical role in engineering flexoelectric materials for practical applications. For instance, in energy harvesting, nanoindentation-assisted studies help optimize the microstructure’s material compositions and mechanical response to enhance flexoelectric performance.^18,149^ Similarly, in functional coatings and electronic interfaces, nanoindentation enables the assessment of mechanical reliability and can provide a flexoelectric response under realistic operating conditions. The combination of nanoindentation with high-resolution imaging techniques such as atomic force microscopy (AFM) and scanning probe microscopy (SPM) further enhances its capabilities, allowing for spatially resolved measurements of flexoelectric phenomena, especially when a large range of strain gradients can be achieved by their combination .

**Figure 16:**
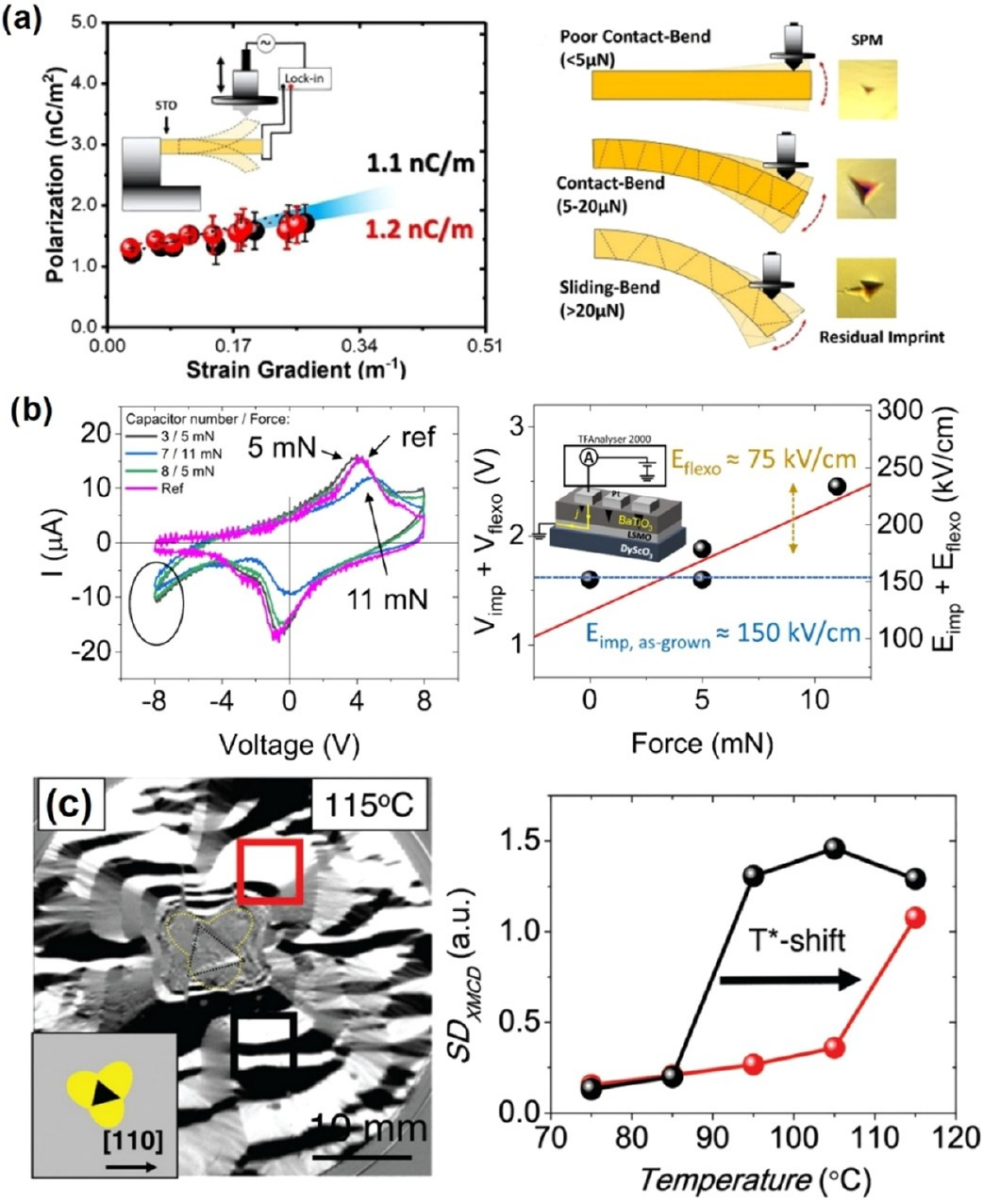
(a) (Left panel) Flexoelectric polarization for STO cantilever beam, measured from both sides of the beam. Red and black dots represent measurements collected from a single side. Dashed lines represent linear fit, accompanied by the total polarization for each measurement, blue region shows the extrapolation of measurements. (Right panel) Schematic of the different initial contact bending scenarios and their associated residual imprint in flat beams, showing the important of contact load. Reused with permission from ref 147, Copyright 2020, Elsevier. (b) (Left panel) *I*–*V* curves of different indented capacitors of BTO films (not short-circuited). (Right panel) *V*_imp_ + *V*_flexo_ and *E*_imp_ + *E*_flexo_ vs indentation load. Reused with permission of AIP from ref 150 Available under a CC-BY 4.0 license. Copyright 2025 Coy, E.; Załęski, K., et al. (c) (Left panel) magnetic domain images of the FeRh film region with a 300 mN indentation measured at 105 °C. Insets show schematic representations of the image symmetry and indent. (Right panel) shows the magnetic contrast of the XMCD images (SDXMCD) of the regions enclosed in red (near indentation) and black (far from the indentation). Reused with permission of the RSC from ref 151 Available under a CC-BY 4.0 license. Copyright 2020, Foerster, Dalmau, Coy et al.

Recent research has also demonstrated that flexoelectricity can be harnessed to modulate photovoltaic response in ferroelectric devices. By employing microscopic indentation on BaTiO3-based capacitor devices, studies have shown that internal electric fields can be controlled via flexoelectric effects, leading to tunable photovoltaic responses.^150^ This approach broadens the scope of flexoelectric applications and opens new pathways for enhancing energy conversion efficiency in photovoltaic systems, in postprocessing indentation, or *ex situ* manipulation of dielectric devices (Figure 16b).

In addition to flexoelectricity, metamagnetism—the transition between antiferromagnetic and ferromagnetic states, has also been influenced by mechanical strain gradients. Research on FeRh alloys has revealed that nanoindentation can induce localized strain patterns, effectively modifying the metamagnetic transition temperature (T*). By generating compressive stresses at selected regions, nanoindentation can enhance the stability of the antiferromagnetic phase, paving the way for novel memory architectures and anti-Ferro-magnetic-spintronic devices.^151^ The ability to manipulate phase transitions via nanoindentation-driven strain nanopatterning has significant implications for magnetic storage and spintronic technologies. This technique enables precise control over the magnetic properties of FeRh films, providing an alternative to traditional methods such as chemical doping or applied magnetic fields.^151^ It is crucial that by relying on mechanical nanopatterning, researchers have demonstrated localized control over magnetic states, leading to the design of high-density storage devices and tunable electronic components (Figure 16c).

As research in flexoelectricity and metamagnetism advances, nanoindentation continues to be a key technique for exploring new materials, refining theoretical models, and guiding the development of next-generation electronic and spintronic devices. Its ability to precisely control mechanical stress and measure resulting electrical and magnetic signals makes it indispensable in pursuing novel applications ranging from microelectronics to bioinspired actuators and adaptive materials.

#### 2.4.2 Other Noncontact Techniques—Brillouin Light Scattering

There are several applications in which nanoindentation can be complemented by other noncontact techniques, such as Raman Spectroscopy, which has already shown large applicability in determining the mechanical properties of 2D materials.^152,153^ However, another direct perspective technique is also based on light scattering. Nanoindentation can be complementary to Brillouin light scattering (BLS). The latter is a contactless, nondestructive technique based on the inelastic scattering of light by the acoustic phonons. It has been used for more than half a century to determine the elastic properties of various materials, including bulk crystals,^154^ supported films,^155,156^ and free-standing membranes.^157,158^ The elastic tensor defines the elastic properties of linear elastic material. Depending on the symmetry of a material, the elastic tensor has a different number of independent elastic constants. According to linear elasticity, the general anisotropic material has 21 independent elastic constants, and the higher the symmetry, the simpler the structure of the elastic tensor.^159,160^ Elastic tensor of crystals with hexagonal symmetry, for instance, has five nonzero independent elastic constants, namely C_11_, C_12_, C_13_, C_33_, C_44_, and one that is given as C_66_ = 1/2 (C_11_–C_12_).^161^ Due to the relation of elastic constants to the phase velocities of acoustic waves propagating in the material, BLS allows their determination. Many papers in the literature report several elastic constants measured directly by BLS in different geometries for materials with various symmetries.^161,162^ For instance, a recent study of hexagonal single-crystal MoSe_2_ membranes obtained elastic constants C_11_ and C_12_ directly from the BLS measurements in backscattering geometry.^161^ However, due to the selection rules,^163^ not all elastic constants are accessible directly by BLS. Nevertheless, to complete the full set of elastic constants, authors typically apply indirect methods such as fitting dispersion relations to get simultaneously missing elastic constants..^164–169^ Here, nanoindentation could help, especially in the case of 2D materials, since the C_33_, or the out-of-plane modulus *E*_*z*_, can, in principle, be experimentally extracted by indentation measurements, allowing BLS to compliment the rest of the elastic constants.

## 3 Conclusions

This review provided a historical and technical overview of the mechanical analysis of materials, tracing its evolution from Hooke’s law to modern nanoindentation techniques. It highlighted key developments in the field, addressing the challenges faced in mechanical characterization throughout the 20th century and demonstrating the growing importance of nanoindentation in studying thin films and nanomaterials. Beyond simply answering fundamental questions such as, “How hard is this material?” or “Can my nanomaterial withstand its intended application?”, nanoindentation offers a deeper understanding of mechanical behavior across different length scales. The inherent complexities arising from discrepancies between the mechanical properties of bulk materials and their nanoscale building blocks further emphasize the need for advanced characterization techniques.

Looking ahead, we elaborate on how the future of nanoindentation in materials science will be shaped by advancements in high-throughput and *in situ* techniques. The integration of nanoindentation into high-throughput thin film characterization workflows enables rapid, spatially resolved, and statistically robust mapping of mechanical properties. This approach generates large data sets that facilitate the identification of composition–property relationships and reveal subtle material features influencing elastic modulus and hardness, even within narrow compositional ranges. Such a capability is critical for accelerating the discovery and optimization of functional materials in compositionally complex systems. The integration of nanoindentation with real-time imaging methods, such as SEM, TEM, and synchrotron XRD, will further enhance its ability to probe mechanical behavior under extreme conditions, including high temperatures and electrochemical environments. Additionally, nanoindentation continues to evolve as a key tool for analyzing thin films, nanostructured materials, and functional coatings, offering critical insights into phase transitions, compositional variations, and structural integrity. The expansion of mechanical mapping techniques, coupled with topographic reconstruction, will enable more precise characterization of nanoscale materials, fostering advancements in flexible electronics, wear-resistant coatings, and next-generation energy storage systems. As research progresses, the refinement of methodologies and the growing adoption of combinatorial approaches will further enhance the predictive capabilities of nanoindentation, ensuring that it remains at the forefront of materials characterization.

*In operando* nanoindentation is emerging as a powerful tool for probing chemomechanical behavior during dynamic electrochemical processes, offering valuable insights for the development of sustainable energy materials. *In operando* indentation demonstrates the capability to characterize the chemomechanical behavior of materials during dynamic electrochemical processes. This approach will aid in unraveling a variety of phenomena in energy materials that involve intimate interactions between mechanics and electrochemistry. The interaction between hydrogen and structural materials, particularly stainless steels, presents various industrial challenges. Understanding hydrogen embrittlement (HE) through *in situ* nanoindentation during electrochemical hydrogen charging is yielding critical advancements in the field, helping to ensure the safety and reliability of structures exposed to hydrogen such as pipelines, storage tanks, and fuel cells.

Despite its significant advancements, the full potential of nanoindentation as a characterization technique has yet to be realized. Future challenges include the mechanical analysis of emerging 2D materials, ultrahard films, and materials subjected to extreme environments. Additionally, as new materials are developed, the search for materials surpassing the mechanical properties of currently known superhard substances will continue. Given the remarkable progress of the past two centuries, it is reasonable to anticipate that the next few decades will bring equally exciting breakthroughs in the field of nanomechanics, expanding both fundamental knowledge and technological applications.

## Footnotes

1 To this date, no student has ever attempted to bite the table. Readers’ experiences might differ.

2 The authors decided to mention Meyer’s and Brinell’s as one single method. However, for historical accuracy, please notice both methods are patent-protected and are still in use today in some industries. Meyer’s method originated in Germany and Brinells’s in Sweden. Due to their similarities and relatively easy conversion of values between methods, they are presented together.

3 The article “Oliver, W. C., & Pharr, G. M. (1992) Journal of materials research, 7(6), 1564–1583.” has over 24,000 citations from specialized books and journals (Scopus—Consulted March 2025). It is considered one of the most cited articles in Materials Sciences to this day.

## References

[1] Young, T. A Course of Lectures on Natural Philosophy and the Mechanical Arts; Printed for J. Johnson: London, 1807.

[2] Poisson, S. D. TRAITÉ DE MÉCANIQUE.: TOME SECOND; Chez Mme veuve Courcier, Imprimeur-Libraire pour les Mathématiques, quai des Augustins, n° 57. 1811.

[3] Greaves G.
N. (2013). Poisson’s
Ratio over Two Centuries: Challenging Hypotheses. Notes Rec. R. Soc. J. Hist. Sci..

[4] Fischer-Cripps, C. Introduction to Contact Mechanics; Springer, 2013; Vol. 53.

[5] Calvert F. C., Johnson R. (1859). On the Hardness of Metals and Alloys. J. Franklin Inst..

[6] Hutchings I. M. (2009). The Contributions
of David Tabor to the Science of Indentation Hardness. J. Mater. Res..

[7] Committee, A. S. M. . ASM Ready Reference: Properties and Units for Engineering Alloys; ASM International, 1997.

[8] Sharpe, W. N. Springer Handbook of Experimental Solid Mechanics; Springer, 2008.

[9] Sneddon I.
N. (1965). The Relation
between Load and Penetration in the Axisymmetric Boussinesq Problem
for a Punch of Arbitrary Profile. Int. J. Eng.
Sci..

[10] Berkovich E. S. (1951). Three Faceted
Diamond Pyramid for Micro-Hardness Testing. Ind. Diamond Rev..

[11] Zak S., Trost C. O. W., Kreiml P., Cordill M. J. (2022). Accurate Measurement
of Thin Film Mechanical Properties Using Nanoindentation. J. Mater. Res..

[12] Li X., Bhushan B. (2002). A Review of Nanoindentation
Continuous Stiffness Measurement
Technique and Its Applications. Mater. Charact..

[13] Mukhopadhyay N. K., Paufler P. (2006). Micro- and Nanoindentation Techniques for Mechanical
Characterisation of Materials. Int. Mater. Rev..

[14] Oliver W. C., Pharr G. M. (1992). Improved Technique for Determining Hardness and Elastic
Modulus Using Load and Displacement Sensing Indentation Experiments. J. Mater. Res..

[15] Duarte M. J., Fang X., Rao J., Krieger W., Brinckmann S., Dehm G. (2021). In Situ Nanoindentation
during Electrochemical Hydrogen Charging:
A Comparison between Front-Side and a Novel Back-Side Charging Approach. J. Mater. Sci..

[16] Warren O. L., Wyrobek T. J. (2005). Nanomechanical Property
Screening of Combinatorial
Thin-Film Libraries by Nanoindentation. Meas.
Sci. Technol..

[17] Hintsala E. D., Hangen U., Stauffer D. D. (2018). High-Throughput
Nanoindentation for
Statistical and Spatial Property Determination. JOM.

[18] Rossi E., Wheeler J. M., Sebastiani M. (2023). High-Speed
Nanoindentation Mapping:
A Review of Recent Advances and Applications. Curr. Opin. Solid State Mater. Sci..

[19] Lotze G., Iyer A. H. S., Bäcke O., Kalbfleisch S., Colliander M. H. (2024). In Situ Characterization of Stresses,
Deformation and
Fracture of Thin Films Using Transmission X-Ray Nanodiffraction Microscopy. J. Synchrotron Radiat..

[20] Gianola D. S., Sedlmayr A., Mnig R., Volkert C. A., Major R. C., Cyrankowski E., Asif S. A. S., Warren O. L., Kraft O. (2011). In Situ Nanomechanical
Testing in Focused Ion Beam and Scanning Electron Microscopes. Rev. Sci. Instrum..

[21] Minor, A. M. In-Situ Nanoindentation in the Transmission Electron Microscope. In In-Situ Electron Microscopy: Applications in Physics, Chemistry and Materials Science; Wiley, 2012; pp 255–277.

[22] Fischer-Cripps, A. C. The IBIS Handbook of Nanoindentation; Fischer-Cripps Laboratories Pty Ltd, 2009.

[23] Fischer-Cripps, A. C. Nanoindentation Instrumentation; Springer, 2011.

[24] Wang S., Shan Z., Huang H. (2017). The Mechanical
Properties of Nanowires. Adv. Sci..

[25] Carlton C. E., Ferreira P. J. (2012). In Situ TEM Nanoindentation
of Nanoparticles. Micron.

[26] Qi H. J., Teo K. B. K., Lau K. K. S., Boyce M. C., Milne W. I., Robertson J., Gleason K. K. (2003). Determination of Mechanical Properties
of Carbon Nanotubes and Vertically Aligned Carbon Nanotube Forests
Using Nanoindentation. J. Mech. Phys. Solids.

[27] Rosenhek-Goldian I., Cohen S. R. (2023). Some Considerations in Nanoindentation
Measurement
and Analysis by Atomic Force Microscopy. J.
Vac. Sci. Technol., A.

[28] Bhushan, B. Nanotribology and Nanomechanics II; Springer: Berlin, Heidelberg, 2011; Vol. 2.

[29] Zhang S., Sun D., Fu Y., Du H. (2005). Toughness Measurement of Thin Films:
A Critical Review. Surf. Coat. Technol..

[30] Leyland A., Matthews A. (2004). Design Criteria for
Wear-Resistant Nanostructured and
Glassy-Metal Coatings. Surf. Coat. Technol..

[31] Leyland A., Matthews A. (2000). On the Significance of the H/E Ratio in Wear Control :
A Nanocomposite Coating Approach to Optimised Tribological Behaviour. Wear.

[32] Tabor, D. The Hardness of Metals; Oxford University Press, 1951.

[33] Greenwood J. A., Williamson J. B. P. (1966). Contact
of Nominally Flat Surfaces. Proc. R. Soc. London,
Ser. A: Math. Phys. Sci..

[34] Tsui T. Y., Pharr G. M., Oliver W. C., Bhatia C. S., White R. L., Anders S., Anders A., Brown I. G. (1995). Nanoindentation
and Nanoscratching of Hard Carbon Coatings for Magnetic Disks. MRS Proc..

[35] Johnson K. L., Keer L. M. (1986). Contact Mechanics. J. Tribol..

[36] Blažek J., Musil J., Stupka P., Čerstvý R., Houška J. (2011). Properties
of Nanocrystalline Al–Cu–O
Films Reactively Sputtered by DC Pulse Dual Magnetron. Appl. Surf. Sci..

[37] Musil J., Jílek R., Meissner M., Tölg T., Čerstvý R. (2012). Two-Phase
Single Layer Al-O-N Nanocomposite
Films with Enhanced Resistance to Cracking. Surf. Coat. Technol..

[38] Musil J., Sklenka J., Čerstvý R., Suzuki T., Mori T., Takahashi M. (2012). The Effect
of Addition of Al in ZrO
2 Thin Film on Its Resistance to Cracking. Surf.
Coat. Technol..

[39] Musil J., Jílek R., Čerstvý R. (2014). Flexible Ti-Ni-N Thin
Films Prepared by Magnetron Sputtering. J. Mater.
Sci. Eng. A.

[40] Chen X., Du Y., Chung Y. W. (2019). Commentary on Using H/E and H3/E2 as Proxies for Fracture
Toughness of Hard Coatings. Thin Solid Films.

[41] Beake B. D., Bell G. A., Goodes S. R., Pickford N. J., Smith J. F. (2010). Improved
Nanomechanical Test Techniques for Surface Engineered Materials. Surf. Eng..

[42] Beake B. D. (2022). The Influence
of the H/E Ratio on Wear Resistance of Coating Systems – Insights
from Small-Scale Testing. Surf. Coat. Technol..

[43] Kumar A., Li D. Y. (2022). Can the H/E Ratio
Be Generalized as an Index for the Wear Resistance
of Materials?. Mater. Chem. Phys..

[44] Deng J., Wu F., Lian Y., Xing Y., Li S. (2012). Erosion Wear of CrN,
TiN, CrAlN, and TiAlN PVD Nitride Coatings. Int. J. Refract. Met. Hard Mater..

[45] Hassani S., Bielawski M., Beres W., Martinu L., Balazinski M., Klemberg-sapieha J. E. (2008). Predictive Tools for the Design of
Erosion Resistant
Coatings. Surf. Coat. Technol..

[46] Musil J., Kunc F., Zeman H., Poláková H. (2002). Relationships
between Hardness, Young’s Modulus and Elastic Recovery in Hard
Nanocomposite Coatings. Surf. Coat. Technol..

[47] Veprek S., Argon A. S. (2002). Towards the Understanding of Mechanical
Properties
of Super- and Ultrahard Nanocomposites. J. Vac.
Sci. Technol., B:Microelectron. Nanometer Struct.--Process., Meas.,
Phenom..

[48] Vepřek S., Haussmann M., Reiprich S., Shizhi L., Dian J. (1996). Novel Thermodynamically
Stable and Oxidation Resistant Superhard Coating Materials. Surf. Coat. Technol..

[49] Musil J. (2012). Hard Nanocomposite
Coatings: Thermal Stability, Oxidation Resistance and Toughness. Surf. Coat. Technol..

[50] Daniel R., Meindlhumer M., Baumegger W., Zalesak J., Sartory B., Burghammer M., Mitterer C., Keckes J. (2017). Grain Boundary Design
of Thin Films: Using Tilted Brittle Interfaces for Multiple Crack
Deflection Toughening. Acta Mater..

[51] Pei Y. T., Galvan D., De Hosson J. T. M. (2005). Nanostructure
and Properties of TiC/a-C:H
Composite Coatings. Acta Mater..

[52] Korsunsky A. M., McGurk M. R., Bull S. J., Page T. F. (1998). On the Hardness
of Coated Systems. Surf. Coat. Technol..

[53] Coy E., Yate L., Kabacińska Z., Jancelewicz M., Jurga S. (2016). Topographic Reconstruction and Mechanical Analysis of Atomic Layer
Deposited Al 2 O 3/TiO 2 Nanolaminates by Nanoindentation. Mater. Des..

[54] Bhushan B., Li X. (1997). Micromechanical and
Tribological Characterization of Doped Single-Crystal
Silicon and Polysilicon Films for Microelectromechanical Systems Devices. J. Mater. Res..

[55] Guo J., Wang H., Meng F., Liu X., Huang F. (2013). Tuning the
H/E * Ratio and E * of AlN Coatings by Copper Addition. Surf. Coat. Technol..

[56] Coy E., Graczyk P., Yate L., Załęski K., Gapiński J., Kuświk P., Mielcarek S., Stobiecki F., Mróz B., Ferrater C., Jurga S. (2017). Second Harmonic
Generation Response in Thermally Reconstructed Multiferroic B′-
Gd2­(MoO4)­3 Thin Films. Sci. Rep..

[57] Megumi K., Yumoto H., Ashida S., Akiyama S., Furuhata Y. (1974). Phase Equilibrium
Diagram for the System Gd2O3-MoO3. Mater. Res.
Bull..

[58] Aizu K., Kumada A., Yumoto H., Ashida S. (1969). Simultaneous
Ferroelectricity
and Ferroelasticity of Gd 2 (MoO 4) 3. J. Phys.
Soc. Jpn..

[59] Ko S. W., Mourey D. A., Clark T., Trolier-McKinstry S. (2010). Synthesis,
Characterization, and Dielectric Properties of β-Gd2­(MoO4)­3
Thin Films Prepared by Chemical Solution Deposition. J. Sol–Gel Sci. Technol..

[60] Suzuki F., Honma T., Komatsu T. (2014). Unique Crystal
Growth with Crystal
Axis Rotation in Multi-Ferroic B′-(Sm,Gd)­2­(MoO4)­3 Narrow Lines
Patterned by Lasers in Glass. J. Phys. Chem.
Solids.

[61] Honma T., Tsukada Y., Komatsu T. (2010). Two-Dimensional Raman
Imaging for
Periodic Domain Structures in Laser-Patterned Ferroelastic B′-(Sm,Gd)­2­(MoO4)­3
Crystal Lines in Glass. Opt. Mater..

[62] Tsukada Y., Honma T., Komatsu T. (2009). Self-Organized
Periodic Domain Structure
for Second Harmonic Generations in Ferroelastic B′-(Sm,Gd)­2­(MoO4)­3
Crystal Lines on Glass Surfaces. Appl. Phys.
Lett..

[63] Haque M. T., Nakajima N., Watanabe K., Kamegashira N., Itoh M. (2003). Structure, Magnetic and Thermal Properties of Gd2MnTiO6. Mater. Chem. Phys..

[64] Xiang X. D., Sun X., Briceño G., Lou Y., Wang K. A., Chang H., Wallace-Freedman W. G., Chen S. W., Schultz P. G. (1995). A Combinatorial
Approach to Materials Discovery. Science.

[65] Hanak J. J. (1970). The ″
Multiple-Sample Concept ″ in Materials Research : Synthesis,
Compositional Analysis and Testing of Entire Multicomponent Systems. J. Mater. Sci..

[66] Zhao J. C. (2006). Combinatorial
Approaches as Effective Tools in the Study of Phase Diagrams and Composition-Structure-Property
Relationships. Prog. Mater. Sci..

[67] Patidar J., Pshyk O., Thorwarth K., Sommerhäuser L., Siol S. (2025). Low Temperature Deposition of Functional
Thin Films on Insulating
Substrates Enabled by Selective Ion Acceleration Using Synchronized
Floating Potential HiPIMS. Nat. Commun..

[68] Pshyk O. V., Zhuk S., Patidar J., Wieczorek A., Sharma A., Michler J., Cancellieri C., Stevanovic V., Siol S. (2025). Discovering Stable Amorphous Ceramics :
From Computational Prediction to Thin-Film Synthesis. Adv. Mater..

[69] Löbel R., Thienhaus S., Savan A., Ludwig A. (2008). Combinatorial
Fabrication
and High-Throughput Characterization of a Ti-Ni-Cu Shape Memory Thin
Film Composition Spread. Mater. Sci. Eng., A.

[70] Ludwig A. (2019). Discovery
of New Materials Using Combinatorial Synthesis and High-Throughput
Characterization of Thin-Film Materials Libraries Combined with Computational
Methods. npj Comput. Mater..

[71] Zhuk S., Wieczorek A., Sharma A., Patidar J., Thorwarth K., Michler J., Siol S. (2023). Combinatorial Reactive Sputtering
with Auger Parameter Analysis Enables Synthesis of Wurtzite Zn2TaN3. Chem. Mater..

[72] Zhuk S., Kistanov A. A., Boehme S. C., Ott N., Mattina F. La., Stiefel M., Kovalenko M. V., Siol S. (2021). Synthesis and Characterization
of the Ternary Nitride Semiconductor Zn2VN3: Theoretical Prediction,
Combinatorial Screening, and Epitaxial Stabilization. Chem. Mater..

[73] Green M. L., Takeuchi I., Hattrick-Simpers J. R. (2013). Applications
of High Throughput (Combinatorial)
Methodologies to Electronic, Magnetic, Optical, and Energy-Related
Materials. J. Appl. Phys..

[74] Boettcher A., Haase G., Thun R. (1955). Strukturuntersuchung
von Mehrstoffsystemen
Durch Kınematische Elektronenbeugung. Int. J. Mater. Res..

[75] Zhao J. C., Jackson M. R., Peluso L. A., Brewer L. N. (2002). A Diffusion
Multiple
Approach for the Accelerated Design of Structural Materials. MRS Bull..

[76] Thomas K., Taylor A. A., Raghavan R., Chawla V., Spolenak R., Michler J. (2017). Microstructure and Mechanical Properties of Metastable
Solid Solution Copper-tungsten Films. Thin Solid
Films.

[77] Miyagawa T., Sakai Y., Mori K., Kato N., Yonezu A., Ishibashi K. (2022). Distribution
of the Mechanical Properties of Ti–Cu
Combinatorial Thin Film Evaluated Using Nanoindentation Experiments
and Molecular Dynamics with a Neural Network Potential. Mater. Today Commun..

[78] Mao F., Taher M., Kryshtal O., Kruk A., Czyrska-Filemonowicz A., Ottosson M., Andersson A. M., Wiklund U., Jansson U. (2016). Combinatorial
Study of Gradient Ag-Al Thin Films: Microstructure, Phase Formation,
Mechanical and Electrical Properties. ACS Appl.
Mater. Interfaces.

[79] Yao J. H., Hostert C., Music D., Frisk A., Björck M., Schneider J. M. (2012). Synthesis
and Mechanical Properties of Fe-Nb-B Thin-Film
Metallic Glasses. Scr. Mater..

[80] Wang Z. T., Zeng K. Y., Li Y. (2011). The Correlation
between Glass Formation
and Hardness of the Amorphous Phase. Scr. Mater..

[81] Wieczerzak K., Groetsch A., Pajor K., Jain M., Müller A. M., Vockenhuber C., Schwiedrzik J., Sharma A., Klimashin F. F., Michler J. (2023). Unlocking the Potential of CuAgZr Metallic Glasses:
A Comprehensive Exploration with Combinatorial Synthesis, High-Throughput
Characterization, and Machine Learning. Adv.
Sci..

[82] Comby-Dassonneville S., Venot T., Borroto A., Longin E., Der Loughian C., Ter Ovanessian B., Leroy M. A., Pierson J. F., Steyer P. (2021). ZrCuAg Thin-Film
Metallic Glasses: Toward Biostatic Durable Advanced Surfaces. ACS Appl. Mater. Interfaces.

[83] Zarnetta R., Kneip S., Somsen C., Ludwig A. (2011). High-Throughput Characterization
of Mechanical Properties of Ti-Ni-Cu Shape Memory Thin Films at Elevated
Temperature. Mater. Sci. Eng., A.

[84] Dwivedi A., Wyrobek T. J., Warren O. L., Hattrick-Simpers J., Famodu O. O., Takeuchi I. (2008). High-Throughput Screening of Shape
Memory Alloy Thin-Film Spreads Using Nanoindentation. J. Appl. Phys..

[85] Hsu S. Y., Lai Y. T., Chang S. Y., Tsai S. Y., Duh J. G. (2022). Combinatorial
Synthesis of Reactively Co-Sputtered High Entropy Nitride (HfNbTiVZr)­N
Coatings: Microstructure and Mechanical Properties. Surf. Coat. Technol..

[86] Keil T., Utt D., Bruder E., Stukowski A., Albe K., Durst K. (2021). Solid Solution
Hardening in CrMnFeCoNi-Based High Entropy Alloy Systems Studied by
a Combinatorial Approach. J. Mater. Res..

[87] Han S. M., Shah R., Banerjee R., Viswanathan G. B., Clemens B. M., Nix W. D. (2005). Combinatorial
Studies of Mechanical
Properties of Ti-Al Thin Films Using Nanoindentation. Acta Mater..

[88] Thienhaus S., Naujoks D., Pfetzing-Micklich J., König D., Ludwig A. (2014). Rapid Identification of Areas of
Interest in Thin Film
Materials Libraries by Combining Electrical, Optical, X-Ray Diffraction,
and Mechanical High-Throughput Measurements: A Case Study for the
System Ni-Al. ACS Comb. Sci..

[89] Wolff-Goodrich S., Marshal A., Pradeep K. G., Dehm G., Schneider J. M., Liebscher C. H. (2021). Combinatorial
Exploration of B2/L21 Precipitation Strengthened
AlCrFeNiTi Compositionally Complex Alloys. J.
Alloys Compd..

[90] Schoeppner R., Ferguson C., Pethö L., Guerra-Nuñez C., Taylor A. A., Polyakov M., Putz B., Breguet J. M., Utke I., Michler J. (2020). Interfacial Adhesion
of Alumina Thin
Films over the Full Compositional Range of Ternary Fcc Alloy Films:
A Combinatorial Nanoindentation Study. Mater.
Des..

[91] Ling J., Wen Z., Yang G., Wang Y., Chen W. (2021). A CALPHAD-Type Young’s
Modulus Database of Ti-Rich Ti–Nb–Zr–Mo System. Calphad.

[92] Warren O. L., Shan Z., Asif S. A. S., Stach E. A., Morris J. W., Minor A. M. (2007). In Situ Nanoindentation
in the TEM. Mater. Today.

[93] Nowak J. D., Rzepiejewska-Malyska K. A., Major R. C., Warren O. L., Michler J. (2010). In-Situ Nanoindentation
in the SEM. Mater. Today.

[94] Zeilinger A., Todt J., Krywka C., Müller M., Ecker W., Sartory B., Meindlhumer M., Stefenelli M., Daniel R., Mitterer C., Keckes J. (2016). In-Situ Observation
of Cross-Sectional Microstructural Changes and Stress Distributions
in Fracturing TiN Thin Film during Nanoindentation. Sci. Rep..

[95] Tadayon K., Bar-On B., Günther B., Vogel C., Zlotnikov I. (2023). In Situ Nanoindentation
at Elevated Humidities. Adv. Mater. Interfaces.

[96] Zeiler S., Jelinek A. S., Terziyska V., Schwaiger R., Mitterer C., Brinckmann S., Maier-Kiener V. (2024). A New Approach
for in Situ Electrochemical Nanoindentation: Side Charging as a Promising
Alternative. Acta Mater..

[97] Wang B., Zhang Z., Cui J., Jiang N., Lyu J., Chen G., Wang J., Liu Z., Yu J., Lin C., Ye F., Guo D. (2017). In Situ TEM
Study of Interaction
between Dislocations and a Single Nanotwin under Nanoindentation. ACS Appl. Mater. Interfaces.

[98] Nanoindentation combined with nanoprobing. https://imina.ch/en/applications/nanoindentation-combined-nanoprobing.

[99] Sun, L. ; Xu, T. ; Zhang, Z. In-Situ Transmission Electron Microscopy; Springer, 2023.

[100] Nili H., Kalantar-Zadeh K., Bhaskaran M., Sriram S. (2013). In Situ Nanoindentation: Probing
Nanoscale Multifunctionality. Prog. Mater. Sci..

[101] Brugarolas T., Gianola D. S., Zhang L., Campbell G. M., Bassani J. L., Feng G., Lee D. (2014). Tailoring and Understanding
the Mechanical Properties of Nanoparticle-Shelled Bubbles. ACS Appl. Mater. Interfaces.

[102] Jiang S., Yang L., Ma X., Zhang H., Guo S., Ren H., Yin W., He X. (2024). Fracture Mechanisms
and Crack Propagation in Monolayer Ti3C2Tx under Nanoindentation:
The Influence of Surface Terminations and Vacancy Defects. ACS Appl. Mater. Interfaces.

[103] Hines R., Hajilounezhad T., Love-Baker C., Koerner G., Maschmann M. R. (2020). Growth
and Mechanics of Heterogeneous,
3D Carbon Nanotube Forest Microstructures Formed by Sequential Selective-Area
Synthesis. ACS Appl. Mater. Interfaces.

[104] Xu Y. N., Liu M. N., Wang M. C., Oloyede A., Bell J. M., Yan C. (2015). Nanoindentation
Study of the Mechanical
Behavior of TiO2 Nanotube Arrays. J. Appl. Phys..

[105] Cho Y., Minsky H. K., Jiang Y., Yin K., Turner K. T., Yang S. (2018). Shear Adhesion of Tapered Nanopillar
Arrays. ACS Appl. Mater. Interfaces.

[106] Lou L., Paul T., Aguiar B. A., Dolmetsch T., Zhang C., Agarwal A. (2022). Direct Observation of Adhesion and
Mechanical Behavior of a Single Poly­(Lactic- Co-Glycolic Acid) (PLGA)
Fiber Using an In Situ Technique for Tissue Engineering. ACS Appl. Mater. Interfaces.

[107] Suchodolskis A., Feiza V., Stirke A., Timonina A., Ramanaviciene A., Ramanavicius A. (2011). Elastic Properties
of Chemically
Modified Baker’s Yeast Cells Studied by AFM. Surf. Interface Anal..

[108] Mikoliunaite L., Makaraviciute A., Suchodolskis A., Ramanaviciene A., Oztekin Y., Stirke A., Jurkaite G., Ukanis M., Carac G., Cojocaru P., Ramanavicius A. (2011). Atomic Force
Microscopy Study of Living Baker’s Yeast Cells. Adv. Sci. Lett..

[109] Suchodolskis A., Stirke A., Timonina A., Ramanaviciene A., Ramanavicius A. (2011). Baker’s Yeast Transformation
Studies by Atomic
Force Microscopy. Adv. Sci. Lett..

[110] Morkvenaite-Vilkončiene I., Ramanavičiene A., Ramanavičius A. (2013). Atomic Force Microscopy as a Tool
for the Investigation
of Living Cells. Medicina.

[111] Hertz H. (1882). Ueber Die Berührung Fester Elastischer Körper. J. Reine Angew. Math..

[112] Cohen S. R., Kalfon-Cohen E. (2013). Dynamic Nanoindentation
by Instrumented
Nanoindentation and Force Microscopy: A Comparative Review. Beilstein J. Nanotechnol..

[113] Section S. P., Railways B., Centre T., Physics S. (1971). Surface Energy
and the Contact of Elastic Solids. Proc. R.
Soc. London A.: Math. Phys. Sci..

[114] Derjaguin B., Muller V., Toporov Y. (1975). Effect of Contact Deformation
on the Adhesion of Elastic Solids. J. Colloid
Interface Sci..

[115] de Vasconcelos L. S., Xu R., Zhao K. (2017). Operando Nanoindentation:
A New Platform to Measure the Mechanical Properties of Electrodes
during Electrochemical Reactions. J. Electrochem.
Soc..

[116] Barnoush A., Vehoff H. (2006). Electrochemical Nanoindentation:
A New Approach to Probe Hydrogen/Deformation Interaction. Scr. Mater..

[117] Vehoff, H. Hydrogen Related Material Problems. In Topics in Applied Physics; Springer, 2007; Vol. 73, pp 215–278.

[118] Robertson I. M. (1999). The Effect of Hydrogen on Dislocation Dynamics. Eng. Fract. Mech..

[119] Robertson I. M. (1999). Controlled Environment Transmission
Electron Microscopy. MRS Proc..

[120] Epler, E. Mechanische Eigenschaften von Lithiumionen Batterieelektrodenmaterialien Bei Verschiedenen Ladezuständen. 2015.

[121] Kim J., Tasan C. C. (2019). Microstructural
and Micro-Mechanical Characterization
during Hydrogen Charging: An in Situ Scanning Electron Microscopy
Study. Int. J. Hydrogen Energy.

[122] Ebner, A. S. Advanced In-Situ Electrochemical Nanoindentation for Investigating Hydrogen- Materials Interactions, 2021.

[123] Yang B., Vehoff H. (2005). Grain Size Effects on the Mechanical
Properties of Nanonickel Examined by Nanoindentation. Mater. Sci. Eng., A.

[124] Göken M., Kempf M., Bordenet M., Vehoff H. (1999). Nanochemical
Characterizations of Metals and Thin Films. Surf. Interface Anal..

[125] Zamanzade, M. Mechanical and Electrochemical Behavior of Fe3Al-XCr Intermetallics 2014 http://scidok.sulb.uni-saarland.de/volltexte/2014/5937.

[126] Barnoush A., Bies C., Vehoff H. (2009). In Situ Electrochemical
Nanoindentation of FeAl (100) Single Crystal: Hydrogen Effect on Dislocation
Nucleation. J. Mater. Res..

[127] Barnoush A., Asgari M., Johnsen R. (2012). Resolving
the Hydrogen
Effect on Dislocation Nucleation and Mobility by Electrochemical Nanoindentation. Scr. Mater..

[128] Barnoush A., Vehoff H. (2008). Hydrogen Embrittlement
of Aluminum
in Aqueous Environments Examined by in Situ Electrochemical Nanoindentation. Scr. Mater..

[129] Göken M., Kempf M. (2001). Pop-Ins in Nanoindentations—the
initial yield point. Z. Metall..

[130] Barnoush A., Vehoff H. (2022). In Situ Electrochemical
Nanoindentation
of a Nickel (111) Single Crystal: Hydrogen Effect on Pop-in Behaviour. Int. J. Mater. Res..

[131] Barnoush A., Vehoff H. (2008). In Situ Electrochemical Nanoindentation:
A Technique for Local Examination of Hydrogen Embrittlement. Corros. Sci..

[132] Barnoush A., Vehoff H. (2010). Recent Developments in the Study
of Hydrogen Embrittlement: Hydrogen Effect on Dislocation Nucleation. Acta Mater..

[133] Stenerud G., Johnsen R., Olsen J. S., He J., Barnoush A. (2017). Effect of Hydrogen on Dislocation Nucleation in Alloy
718. Int. J. Hydrogen Energy.

[134] Barnoush A., Kheradmand N., Hajilou T. (2015). Correlation between
the Hydrogen Chemical Potential and Pop-in Load during in Situ Electrochemical
Nanoindentation. Scr. Mater..

[135] Wang D., Lu X., Deng Y., Guo X., Barnoush A. (2019). Effect of Hydrogen on Nanomechanical Properties in
Fe-22Mn-0.6C TWIP Steel Revealed by in-Situ Electrochemical Nanoindentation. Acta Mater..

[136] Kheradmand N., Johnsen R., Olsen J. S., Barnoush A. (2016). Effect of
Hydrogen on the Hardness of Different Phases in Super Duplex Stainless
Steel. Int. J. Hydrogen Energy.

[137] Wang D., Lu X., Deng Y., Wan D., Li Z., Barnoush A. (2019). Effect of Hydrogen-Induced Surface
Steps on the Nanomechanical
Behavior of a CoCrFeMnNi High-Entropy Alloy Revealed by in-Situ Electrochemical
Nanoindentation. Intermetallics.

[138] Zhao Y., Lee D. H., Lee J. A., Kim W. J., Han H. N., Ramamurty U., Suh J. Y., Jang J. (2017). il. Hydrogen-Induced
Nanohardness Variations in a CoCrFeMnNi High-Entropy Alloy. Int. J. Hydrogen Energy.

[139] Zhao Y., Lee D. H., Seok M. Y., Lee J. A., Phaniraj M. P., Suh J. Y., Ha H. Y., Kim J. Y., Ramamurty U., Jang J. il. (2017). Resistance of CoCrFeMnNi
High-Entropy
Alloy to Gaseous Hydrogen Embrittlement. Scr.
Mater..

[140] Zhao K., Cui Y. (2016). Understanding the Role of Mechanics
in Energy Materials: A Perspective. Extreme
Mech. Lett..

[141] Khosrownejad S. M., Curtin W. A. (2016). Model for Charge/Discharge-Rate-Dependent
Plastic Flow in Amorphous Battery Materials. J. Mech. Phys. Solids.

[142] Brassart L., Suo Z. (2013). Reactive Flow in Solids. J. Mech. Phys. Solids.

[143] Kuznetsov V., Zinn A. H., Zampardi G., Borhani-Haghighi S., La Mantia F., Ludwig A., Schuhmann W., Ventosa E. (2015). Wet Nanoindentation of the Solid Electrolyte Interphase
on Thin Film Si Electrodes. ACS Appl. Mater.
Interfaces.

[144] de Vasconcelos L. S., Sharma N., Xu R., Zhao K. (2019). In-Situ Nanoindentation
Measurement of Local Mechanical Behavior of a Li-Ion Battery Cathode
in Liquid Electrolyte. Exp. Mech..

[145] Kalinin S. V., Meunier V. (2008). Electronic Flexoelectricity
in Low-Dimensional
Systems. Phys. Rev. B.

[146] Gharbi M., Sun Z. H., Sharma P., White K., El-Borgi S. (2011). Flexoelectric Properties of Ferroelectrics
and the
Nanoindentation Size-Effect. Int. J. Solids
Struct..

[147] Coy E. (2020). Method for Probing Flexoelectric
Response of Free-Standing Cantilever
Beams by Nanometric Oscillations with Nanoindentation Technique. Measurement.

[148] Liang X., Dong H., Wang Y., Ma Q., Shang H., Hu S., Shen S. (2024). Advancements of Flexoelectric
Materials and Their Implementations in Flexoelectric Devices. Adv. Funct. Mater..

[149] Zhang W., Yan X., Meng Y., Zhang C., Youn S.-K., Guo X. (2022). Flexoelectric Nanostructure
Design
Using Explicit Topology Optimization. Comput.
Methods Appl. Mech. Eng..

[150] Coy E., Załęski K., Budziałowski M., Zou J., Dix N., Sánchez F., Fina I. (2025). Modulated Photovoltaic
Response in a Flexoelectric Device Using Microscopic Indentation. APL Mater..

[151] Foerster M., Menéndez E., Coy E., Quintana A., Gómez-Olivella C., de los Ojos D. E., Vallcorba O., Frontera C., Aballe L., Nogués J., Sort J., Fina I. (2020). Local Manipulation
of Metamagnetism
by Strain Nanopatterning. Mater. Horiz..

[152] Akinwande D., Brennan C. J., Bunch J. S., Egberts P., Felts J. R., Gao H., Huang R., Kim J.-S., Li T., Li Y., Liechti K. M., Lu N., Park H. S., Reed E. J., Wang P., Yakobson B. I., Zhang T., Zhang Y.-W., Zhou Y., Zhu Y. (2017). A Review on Mechanics
and Mechanical Properties of 2D Materials—Graphene and Beyond. Extreme Mech. Lett..

[153] Cong X., Liu X.-L., Lin M.-L., Tan P.-H. (2020). Application
of Raman Spectroscopy to Probe Fundamental Properties of Two-Dimensional
Materials. npj 2D Mater. Appl..

[154] Singaraju A. B., Bahl D., Stevens L. L. (2019). Brillouin
Light
Scattering: Development of a Near Century-Old Technique for Characterizing
the Mechanical Properties of Materials. AAPS
PharmSciTech.

[155] Link A., Sooryakumar R., Bandhu R. S., Antonelli G. A. (2006). Brillouin
Light Scattering Studies of the Mechanical Properties of Ultrathin
Low-k Dielectric Films. J. Appl. Phys..

[156] Cheng W., Sainidou R., Burgardt P., Stefanou N., Kiyanova A., Efremov M., Fytas G., Nealey P. F. (2007). Elastic
Properties and Glass Transition of Supported
Polymer Thin Films. Macromolecules.

[157] Sledzinska M., Graczykowski B., Placidi M., Reig D. S., Sachat A. El., Reparaz J. S., Alzina F., Mortazavi B., Quey R., Colombo L., Roche S., Torres C. M. S. (2016). Thermal
Conductivity of MoS 2 Polycrystalline Nanomembranes. 2D Mater..

[158] Zhang X., Sooryakumar R., Bussmann K. (2003). Confinement and Transverse
Standing Acoustic Resonances in Free-Standing Membranes. Phys. Rev. B.

[159] Cowin S. C., Mehrabadi M. M. (1995). Anisotropic Symmetries of Linear
Elasticity. Appl. Mech. Rev..

[160] Zou W.-N., Tang C.-X., Lee W.-H. (2013). Identification
of
Symmetry Type of Linear Elastic Stiffness Tensor in an Arbitrarily
Orientated Coordinate System. Int. J. Solids
Struct..

[161] Babacic V., Reig D. S., Varghese S., Vasileiadis T., Coy E., Tielrooij K., Graczykowski B. (2021). Thickness-Dependent Elastic Softening
of Few-Layer Free-Standing MoSe 2. Adv. Mater..

[162] Djemia P., Roussigné Y., Dirras G. F., Jackson K. M. (2004). Elastic
Properties of β-SiC Films by Brillouin Light Scattering. J. Appl. Phys..

[163] Mutti, P. ; Bottani, C. E. ; Ghislotti, G. ; Beghi, M. ; Briggs, G. A. D. ; Sandercock, J. R. Surface Brillouin Scattering—Extending Surface Wave Measurements to 20 GHz. In Advances in Acoustic Microscopy; Springer US: Boston, MA, 1995; pp 249–300.

[164] Wittkowski T., Jorzick J., Jung K., Hillebrands B. (1999). Elastic Properties
of Thin H-BN Films Investigated by Brillouin Light Scattering. Thin Solid Films.

[165] Fioretto D., Carlotti G., Socino G., Modesti S., Cepek C., Giovannini L., Donzelli O., Nizzoli F. (1995). Brillouin-scattering
determination of the elastic constants of epitaxial fccC60film. Phys. Rev. B.

[166] Every A. G., Sumanya C., Mathe B. A., Zhang X., Comins J. D. (2016). Optimized Determination of Elastic Constants of Crystals
and Their Uncertainties from Surface Brillouin Scattering. Ultrasonics.

[167] Zizka J., King S., Every A. G., Sooryakumar R. (2016). Mechanical
Properties of Low- and High- k Dielectric Thin Films: A Surface Brillouin
Light Scattering Study. J. Appl. Phys..

[168] Lee S., Hillebrands B., Stegeman G. I., Cheng H., Potts J. E., Nizzoli F. (1988). Elastic Properties
of Epitaxial ZnSe(001) Films on
GaAs Measured by Brillouin Spectroscopy. J.
Appl. Phys..

[169] Carlotti G. (2018). Elastic Characterization
of Transparent and Opaque
Films, Multilayers and Acoustic Resonators by Surface Brillouin Scattering:
A Review. Appl. Sci..

